# Chitosan-Based Nanoparticles Against Viral Infections

**DOI:** 10.3389/fcimb.2021.643953

**Published:** 2021-03-17

**Authors:** Homa Boroumand, Fereshteh Badie, Samaneh Mazaheri, Zeynab Sadat Seyedi, Javid Sadri Nahand, Majid Nejati, Hossein Bannazadeh Baghi, Mohammad Abbasi-Kolli, Bita Badehnoosh, Maryam Ghandali, Michael R. Hamblin, Hamed Mirzaei

**Affiliations:** ^1^ School of Medicine, Kashan University of Medical Sciences, Kashan, Iran; ^2^ Department of Microbiology, Faculty of Medicine, Kashan University of Medical Sciences, Kashan, Iran; ^3^ Department of Analytical Chemistry, Faculty of Chemistry, University of Kashan, Kashan, Iran; ^4^ Department of Cell and Molecular Biology, Faculty of Chemistry, University of Kashan, Kashan, Iran; ^5^ Department of Virology, Faculty of Medicine, Iran University of Medical Sciences, Tehran, Iran; ^6^ Anatomical Sciences Research Center, Institute for Basic Sciences, Kashan University of Medical Sciences, Kashan, Iran; ^7^ Department of Microbiology, Faculty of Medicine, Tabriz University of Medical Sciences, Tabriz, Iran; ^8^ Infectious and Tropical Diseases Research Center, Tabriz University of Medical Sciences, Tabriz, Iran; ^9^ Department of Medical Genetics, Faculty of Medical Sciences, Tarbiat Modares University, Tehran, Iran; ^10^ Department of Gynecology and Obstetrics, Dietary Supplements and Probiotic Research Center, Alborz University of Medical Sciences, Karaj, Iran; ^11^ School of Medicine, Iran University of Medical Sciences, Tehran, Iran; ^12^ Laser Research Centre, Faculty of Health Science, University of Johannesburg, Doornfontein, South Africa; ^13^ Research Center for Biochemistry and Nutrition in Metabolic Diseases, Institute for Basic Sciences, Kashan University of Medical Sciences, Kashan, Iran

**Keywords:** viral infection, chitosan, nanoparticles, delivery system, therapy

## Abstract

Viral infections, in addition to damaging host cells, can compromise the host immune system, leading to frequent relapse or long-term persistence. Viruses have the capacity to destroy the host cell while liberating their own RNA or DNA in order to replicate within additional host cells. The viral life cycle makes it challenging to develop anti-viral drugs. Nanotechnology-based approaches have been suggested to deal effectively with viral diseases, and overcome some limitations of anti-viral drugs. Nanotechnology has enabled scientists to overcome the challenges of solubility and toxicity of anti-viral drugs, and can enhance their selectivity towards viruses and virally infected cells, while preserving healthy host cells. Chitosan is a naturally occurring polymer that has been used to construct nanoparticles (NPs), which are biocompatible, biodegradable, less toxic, easy to prepare, and can function as effective drug delivery systems (DDSs). Furthermore, chitosan is Generally Recognized as Safe (GRAS) by the US Food and Drug Administration (U.S. FDA). Chitosan NPs have been used in drug delivery by the oral, ocular, pulmonary, nasal, mucosal, buccal, or vaginal routes. They have also been studied for gene delivery, vaccine delivery, and advanced cancer therapy. Multiple lines of evidence suggest that chitosan NPs could be used as new therapeutic tools against viral infections. In this review we summarize reports concerning the therapeutic potential of chitosan NPs against various viral infections.

## Introduction

Infectious diseases caused by bacteria, fungi, viruses, and parasites are responsible for nearly 15 million deaths globally, of which human immunodeficiency virus (HIV), malaria, tuberculosis, as well as acute respiratory infection including the newly emerged COVID-19 have been considered to be the key causes of death (Panácek et al., 2009; Li et al., 2011; Qasim et al., 2014). Viral infections cause substantial health concerns that affect millions of individuals globally, and negatively impact human health and socio-economic development (Mehendale et al., 2013; Nahand et al., 2020a; Nahand et al., 2020b). Effective treatments of viral infections are limited by increased incidence of drug resistance, particularly related to HIV (Little et al., 2002; Shafer et al., 2007; Lockhat et al., 2016; Maseko et al., 2016) and influenza (Hayden, 2009). Drug resistant viral infections are a public health threat, causing widespread morbidity as well as mortality (Cosgrove, 2006), and additional costs caused by expensive drugs (Li et al., 2011; Qasim et al., 2014). Therefore, there is an urgent need to develop novel approaches to prevent and treat these viral infections.

Nanotechnology is concerned with particles with sizes within the nanometer range from10^−9^ or 1 billionth of a meter (Nalwa, 1999; Parboosing et al., 2012). Nanobiotechnology refers to interactions of nanoscience with biological systems (Scheller et al., 1995; Niemeyer, 2006). Nanomedicine involves the use of nano-structured materials for the diagnosis, prevention, or treatment of diseases (Medepalli, 2008).The first use of nanoparticles (NPs) in medicine was to increase the solubility and stability of drugs with low bioavailability (Schütz et al., 2013). NPs can exert antiviral activity through several mechanisms. Firstly, NPs are useful in viral treatment due to their specific features, such as small particle size (Kumar et al., 2012; Parboosing et al., 2012), high ratio of surface area to volume (McNeil, 2011), and adjustable surface charge (Caron et al., 2010; Petros and DeSimone, 2010). Secondly, it has been shown that NPs can display biomimicry features (Bowman et al., 2008; Sanvicens and Marco, 2008; Gagliardi, 2017), and therefore can exert intrinsic antiviral activity; *e.g.* silver nanoparticles (AgNPs) (Sun et al., 2005; Lara et al., 2010) or dendrimers (Wang et al., 2006; Mallipeddi and Rohan, 2010). Thirdly, NPs can also increase the stability of the loaded drug, allowing dose optimization and more predictable delivery, which all stem from the drug encapsulation ability (Chiappetta et al., 2011; Kumar et al., 2012). NPs can be tailored to create stable structures (Goldberg et al., 2007), or modified by attachment of polyethylene glycol (PEG) (Gabizon et al., 1994; Alexis et al., 2008; McNeil, 2011; Santos-Martinez et al., 2014). Fourthly, engineered NPs can significantly enhance drug delivery by the use of targeting moieties that recognize receptors or biomarkers to increase the specificity for target cells, target tissues, or subcellular compartments (Muthu and Singh, 2009; Petros and DeSimone, 2010; McNeil, 2011).

Chitosan (CH) is a modified biopolymer obtained *via* partial de-acetylation of the naturally occurring polysaccharide called chitin, which contains randomly distributed (1→ 4)-linked N-acetyl glucosamine and glucosamine units (Rashki et al., 2021). CH can be obtained as a white powder, composed of rigid, inflexible, and nitrogen-containing polysaccharide chains of varying length and molecular weight (Badawy and Rabea, 2011). In addition, CH has multi-faceted applications because of its non-toxicity, bio-degradability, and intrinsic antimicrobial properties. CH has been used in biomedical preparations, genetic engineering, agricultural sector, environmental pollution control, food manufacturing, paper manufacturing, photography, and water treatment (Cheba, 2011). CHNPs show interesting interface and surface effects due to their very small size (Ingle et al., 2008). Several studies have suggested that CH NPs could be used as new therapeutic options against viral infections.

## Chitosan (CH) and CH NPs

**Figure 1:**
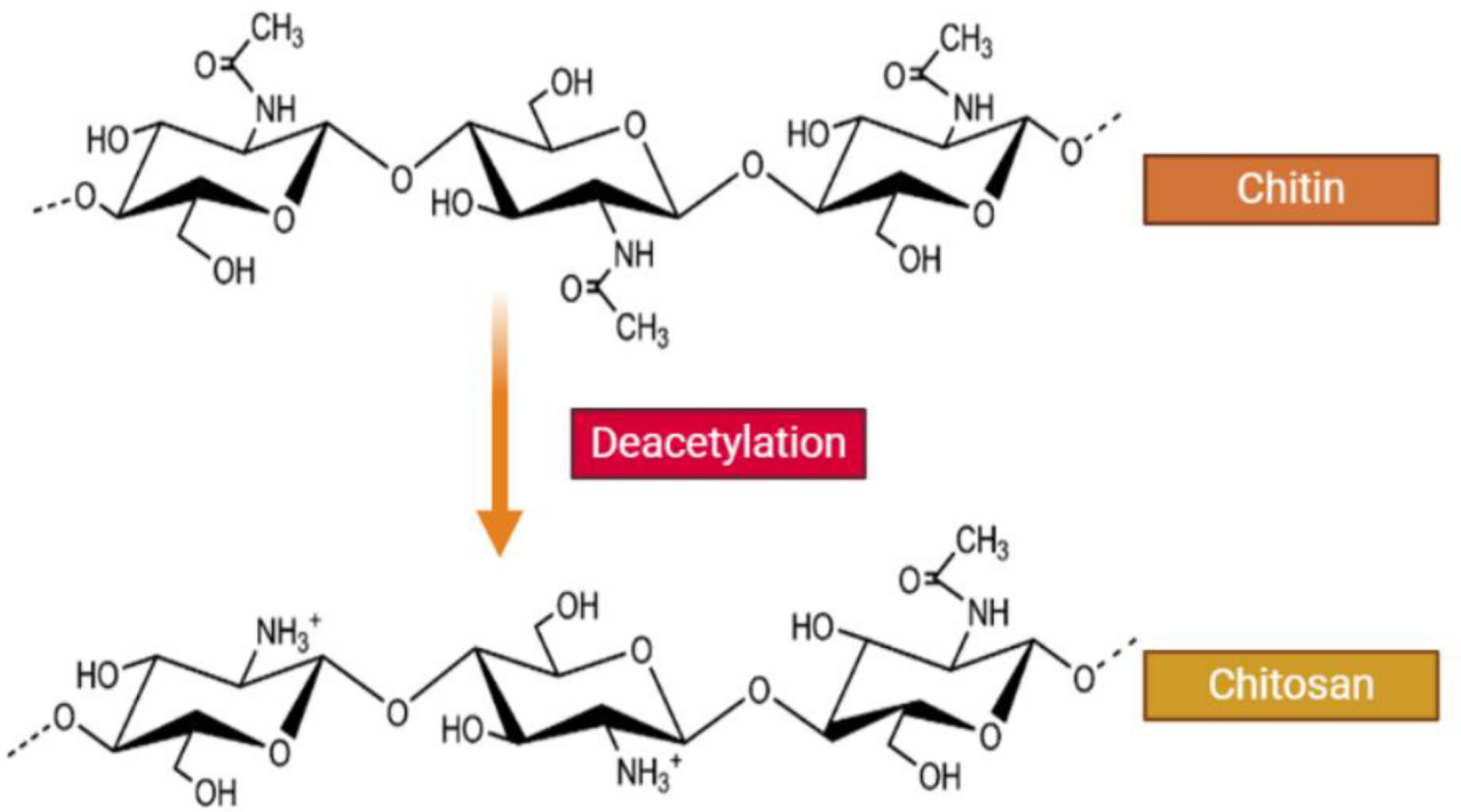
Structure of chitin and chitosan. CH is a naturally occurring water-soluble cationic polysaccharide that is generated by alkaline deacetylation of chitin. CH is a linear polysaccharide composed of β–(1-4) linked 2-acetamide-2-deoxy-D-glucose (a neutral sugar unit GlcNAc or A-unit) and 2-amino 2-deoxy-D-glucose (a cationic sugar unit GlcN or D-unit). CH can have a broad range of degree of deactylation (F_A_), as well as variable chain length and molecular weight.

CH is a straight chain, cationic polysaccharide that is prepared by partial deacetylation of chitin, which is commonly carried out by alkaline hydrolysis (**Figure 1**). CH commonly refers to cationic co-polymers that contain 2 amino 2-deoxy-β-D-glucose residues (60% to 100%) as well as 2 acetamino-2-deoxy-β-D-glucoside residues (0% to 50%), attached together by ß (1→4) bonds (Nasrollahzadeh et al., 2020; Tao et al., 2020). The mean degree of deacetylation is usually >60% (Ng et al., 2002; Varma et al., 2004). The average degree of deacetylation and the molecular weight specifies the total number of amide residues and the number of primary amine residues, and this ratio directly influences the CH solubility and the chemical, biological and physical properties (Kmiec et al., 2017; Jaworska et al., 2020; Rashki et al., 2020; Yang et al., 2020a).

The CH amino groups are more reactive compared to the acetamido-groups of chitin. In fact, the nitrogen free-electron pair in the amino groups can bind to metallic cations, and the average degree of deacetylation determines their number. In addition, protonation of the amino groups in an acidic solution will electrostatically attract surrounding anions (Kmiec et al., 2017).

CH, unlike chitin, can be dissolved in a dilute acidic solution to form a soluble cationic polymer, which has different properties to other polysaccharides (Kmiec et al., 2017). Following a lengthy period of agitation, CH can be dissolved in dilute acids like acetic, formic, lactic, or inorganic acids. Nonetheless, the solubility depends on the degree of deacetylation, molecular weight, bio-polymer concentration, pH, and ionic strength (Varma et al., 2004). In addition to the primary amino groups, CH contains numerous primary and secondary hydroxyl-groups at the C-2, C-3 and C-6 positions, which show robust interactions with water (Peter, 1995).

CH has advantages such as biodegradability, hydrophilicity, biocompatibility, bioactivity, and is obtained from renewable and natural sources. It can be processed into distinct forms, such as solutions, blends, sponges, membrane, gels, paste, tablets, microspheres, or microgranules for different applications (Kumar et al., 2004; Pillai et al., 2009).

Henri Braconnot first discovered chitin in 1811 while researching different mushrooms. Next, Prof. C. Rouget (1859) found that alkaline treatment of chitin produced a material, which could be dissolved in acids, unlike chitin. Hoppe Seiler was the first to call this deacetylated chitin “chitosan” (Badawy and Rabea, 2011). Next to cellulose, chitin is the most widespread biopolymer found in all of the natural world. Chitin is the main component of insect cuticles, yeast, green algae and fungal cell walls (Einbu and Vårum, 2008), and is also found in crab and shrimp shells (Wang and Xing, 2007). On the other hand, chitosan itself is very rare in nature, being only found in the cell walls of certain fungi (Muzzarelli et al., 1986; Yen et al., 2009). There are three principal types of chitin called α, β, and γ. The α-chitin possesses anti-parallel chains, whereas β-chitin possesses parallel chains formed by in-sheet hydrogen bonds. The γ-chitin is made up from combinations of α and β chitin types with both parallel and anti-parallel chain arrangements (Franca et al., 2008; Yen et al., 2009).

## Preparation of CH NPs

The most common procedures for preparing CH-based NPs are, emulsion based solvent evaporation, ionotropic gelation, solvent diffusion, emulsification, as well as the microemulsion method (Garg et al., 2019; Zhang et al., 2019; Ansari et al., 2020). Some of the benefits associated with the above techniques include the use of less organic solvent and the need for a lower force. However, the size and surface charge of the CH NPs prepared *via* the above procedures, depend on the degree of deacetylation and the molecular weight (Mohammed et al., 2017). In addition, hydrophobic interactions, electrostatic interactions, and hydrogen bonding all govern the extent to which drugs will be entrapped inside the polymeric matrix. Moreover, the drug loading and drug release will need to be investigated for each application of CH NPs, considering the physiological environment at the administration site. In fact, both the ionic strength and the presence of enzymes or proteins will affect drug release and stability (e.g., the milieu of the eye versus the GI tract). It is fortunate that there are numerous techniques available to prepare the optimum CH NP formulation for each application (Mohammed et al., 2017).

### Ionotropic Gelation

Calvo et al. were the first to describe the ionotropic gelation method for the preparation of CH NPs. Thereafter, additional experts in the field investigated and developed it further (Calvo et al., 1997). This procedure relies on electrostatic interactions between the amine groups of CH and a variety of polyanions with a negative charge, such as tri-polyphosphate (Divya and Jisha, 2018). First it is necessary to dissolve the CH in acetic acid in the presence or the absence of a stabilizing agent, such as a poloxamer. In the next step, the polyanion is added, and the NPs are spontaneously generated by mechanical shaking at room temperature. It is possible to modify the size as well as the surface charge of the particles by varying the CH-to-stabilizer molar ratio (Divya and Jisha, 2018). Researchers have found an overall increase in the particle compactness and size by increasing the CH concentration and the ratio of polymer-to-polyanion (Jonassen et al., 2012). Results indicated that NPs dispersed in a saline solution showed higher stability and a smaller particle size in the presence of sodium chloride, which may be caused by the monovalent sodium salt screening out the electrostatic repulsion between the positively charged amine groups on the CH backbone. The presence of salt may enhance the flexibility within the polymer chain as well as its stability (Ilium, 1998).

### Polyelectrolyte Complexes (PEC)

A polyelectrolyte complex is formed when plasmid DNA is mixed with a cationic charged polymer such as CH, which self-assembles into nanostructures because the anionic charge on the DNA is neutralized by the cationic polymer (Divya and Jisha, 2018). Spontaneous formation of CH NPs occurs after adding the DNA solution to the CH dissolved in acetic acid solution with mechanical shaking at room temperature (Erbacher et al., 1998).

### Reverse Micelles Preparation

Brunel et al. first described a method for the preparation of reverse micelles (Brunel et al., 2008). A key benefit was the absence of poisonous organic solvents and cross-linkers. Moreover, it was possible to obtain ultra-fine NPs in a narrow size range by this technique (Divya and Jisha, 2018). The CH aqueous solution is poured into an organic solvent containing a surfactant with constant agitation to allow the formation of reverse micelles (Zhao et al., 2011).

### Microemulsion Method

In the microemulsion procedure, CH is mixed with glutaraldehyde in an acetic acid solution and poured into a surfactant in an organic solvent such as hexane (Mohammed et al., 2017). Then the mixture is continuously shaken at room temperature overnight. This covalent cross-linking reaction results in the formation of CH NPs. In the next step, the organic solvent is removed *via* evaporation under vacuum (Mohammed et al., 2017). Excessive surfactant can be removed *via* precipitation with calcium chloride and centrifugation. The resultant NP suspension is finally dialyzed and lyophilized (Maitra et al., 1999). The CH NPs show a very narrow size distribution, which can be controlled by varying the glutaraldehyde concentration (Wang et al., 2008). The disadvantages of the microemulsion method are the need for an organic solvent, a prolonged reaction time, and a complicated washing-step (Nagpal et al., 2010).

### Emulsification and Solvent Diffusion

In order to prepare an oil-in-water emulsion, an aqueous solution of CH and a stabilizer is mixed with an organic solvent by mechanical shaking accompanied by increased pressure homogenization (Niwa et al., 1993; El-Shabouri, 2002). Precipitation is caused by adding a large amount of water into the emulsion, and the NPs are formed with a size ranging between 300 nm and 500 nm. This procedure is a good choice to load hydrophobic molecules, with a high entrapment efficiency (EE%); however, a key caveat is the need for an increased shear force (Mohammed et al., 2017).

## Chitosan as an Adjuvant for Antiviral Vaccines

As the second most plentiful polysaccharide in nature, CH has seen widespread utilization in drug formulation and pharmaceutics, due to its non-toxicity, biodegradability, biocompatibility, and its ability to cross tight epithelial junctions (Ilium, 1998). CH can open tight intercellular junctions, thus allowing the loaded drugs to penetrate better into tissue and be taken up by target cells (Van Der Lubben et al., 2001a; Van der Lubben et al., 2001b; Van der Lubben et al., 2001c). CH NPs are also promising biomaterials to improve antigen delivery and the performance of vaccines. The association of antigens with chitosan-based nanoparticle systems, has shown that antigen uptake by mucosal lymphatic tissues is increased, resulting in more potent mucosal and systemic immune responses to different antigens (Bramwell and Perrie, 2006). Chitosan has mucoadhesive properties, which increases the long-term adhesion of the CH NPs, and therefore a longer time in contact with the bloodstream capillaries, leading to greater uptake of the antigen protein or the plasmid DNA (Khatri et al., 2008a).

The respiratory mucosal surface acts as a primary immune defense barrier, and is the main site of influenza virus infection. Many studies have shown that mucosal vaccines could be used to induce both a systemic and a local mucosal immune response (Yuki and Kiyono, 2003). When vaccines are combined with an adjuvant, the induction of mucosal immunity is improved. Some adjuvants are toxins, such as *Escherichia coli* heat-labile toxin or cholera toxin (Glück et al., 2000). However these toxins can cause acute diarrhea and damage to the central nervous system (CNS) (van Ginkel et al., 2000). Therefore, the development of more effective and safer adjuvants is essential to improve mucosal immunization.

**Figure 2:**
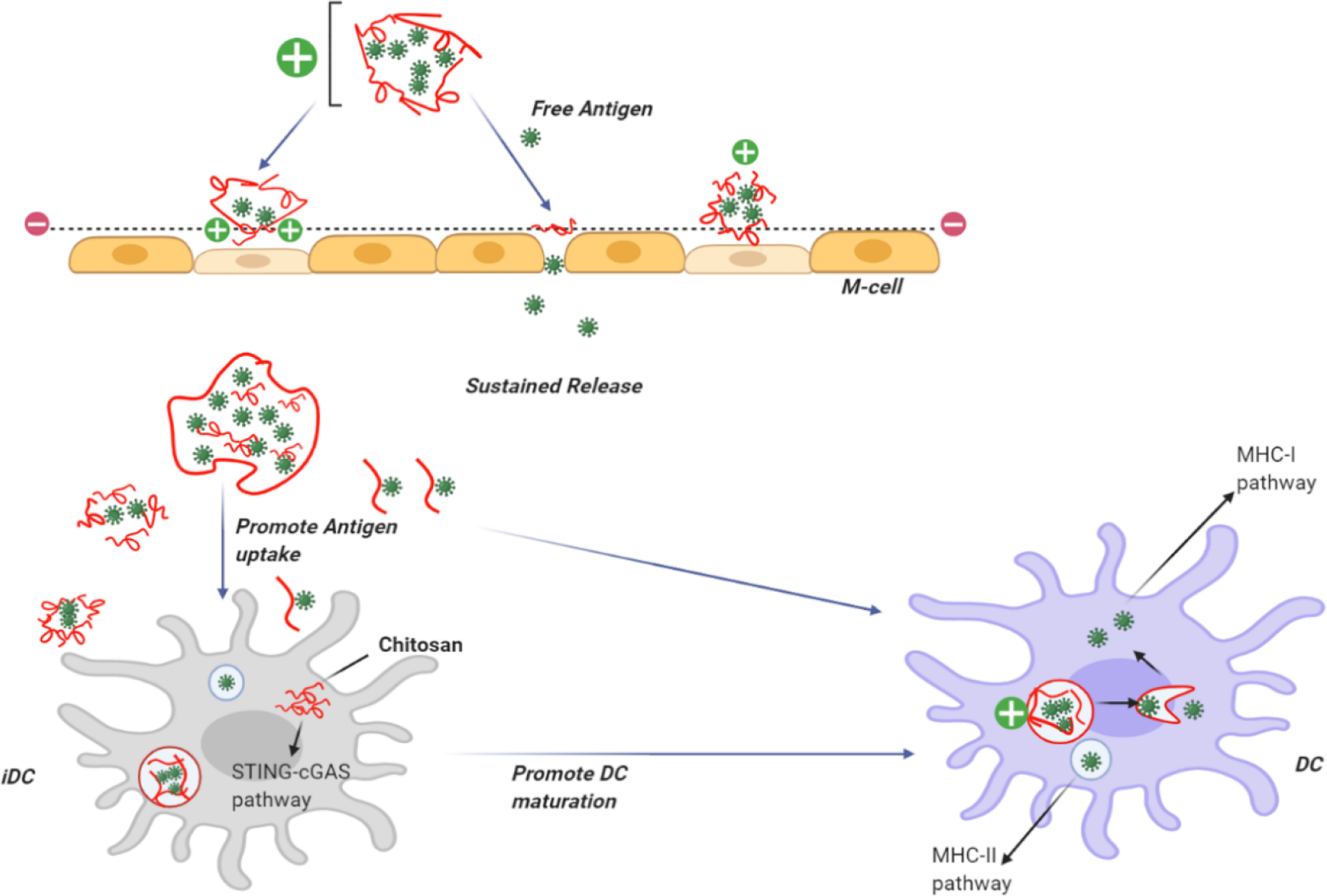
Use of chitosan as an adjuvant for vaccine delivery. CH can be dissolved in acid to provide a positive charge on the particle surface. CH NPs containing antigens can adhere to the cell surface in the nasal mucosa giving a prolonged residence time. CH can open the tight junctions and allow the transfer of free antigens through the para-cellular route, as well as the transcytosis of the packaged antigens *via* M-cells. CH becomes insoluble at physiological pH, providing slow release of encapsulated antigens and uptake bydendritic cells DCs. Following uptake by DCs, CH activates the STING-cGAS pathway resulting in DC maturation. After the insoluble particles have been taken up by cells *via* endocytosis, CH becomes soluble at the acidic pH and the antigens escape from the lysosomes into the cytoplasm for cross presentation through the MHC-I pathway. Antigens taken up by DCs can berouted to the MHC-II pathway.

Recently, the use of self-assembled biodegradable CH NPs has emerged as an efficient vaccine delivery system (Uto et al., 2007). The interaction of NPs with antigens leads to improved antigen-specific acquired immune responses by increasing the uptake by antigen presenting cells, such as dendritic cells (DCs) and macrophages (**Figure 2**) (Akagi et al., 2005).

In addition, when NPs are taken up by DCs, it leads to up-regulation of costimulatory molecules, stimulation of cytokine production, and increased T-cell stimulation (Uto et al., 2007). Therefore, NP delivery systems have been proposed as efficient adjuvants for sub-unit vaccines. Nevertheless, the non-biodegradable nature of many types of NPs is a problem with the delivery of vaccines. Moreover, poly-gamma-glutamic acid (g-PGA), which is a capsular polymer secreted by *Bacillus subtilis*, is considered to be a safer alternative. (Poo et al., 2010). Moon et al. (2012) described a type of hybrid NPs composed of g-PGA plus CH. They showed that the hybrid NPs allowed intranasal immunization by an inactivated virus or by the model antigen, recombinant influenza hemagglutinin protein (rHA) leading to increased anti-HA immunoglobulin A (IgA) and IgG responses. This intranasal vaccination protocol protected mice against infection with a pathogenic dose of influenza type A H5N1 virus (Moon et al., 2012).

Mucosal immunization can lead to a mucosal immune response with or without a systemic immune response. The mucosal response was due to the production of secreted IgA (sIgA) antibody, without the production of systemic immunization (Nugent, 1998). However, it is accepted that oral vaccination shows higher efficiency. Degradation of antigens in the acidic stomach environment as well as enzymatic degradation in the intestinal tract contributes to the higher efficacy of oral vaccination (Gabor et al., 2002; Lavelle et al., 2004; Singh et al., 2004).

Chowdhury et al. described the production of a mucosal influenza vaccine system, which showed broad cross-protection against both emerging and seasonal influenza A viruses. They loaded poly-γ-glutamic acid (γ-PGA)-chitosan NPs (PC NPs) with the strongly conserved matrix protein-2 (sM2), the mucosal adjuvant cholera toxin subunit A1 (CTA1), and a fusion peptide of hemagglutinin (HA_2_), all of which have been demonstrated to be safe natural substances with the ability of being absorbed by mucosal membrane as a mucosal adjuvant (Chowdhury et al., 2017). Results of the study showed that mucosal administration of this sM2HA2CTA1/PC NP preparation could trigger both local immunity (IgA& IgG) at the inoculation site, and remote systemic immunity. In addition, researchers confirmed that the inclusion of sM2 and HA2 triggered specific cell-mediated immune responses. The following challenge tests were carried out in BALB/c mice. IAV strains: A/Puerto Rico/8/34(H1N1), 10 MLD_50_ of A/EM/Korea/W149/06(H5N1), A/Aquatic bird/Korea/W44/2005 (H7N3), A/Chicken/Korea/116/2004(H9N2), and A/Aquatic bird/Korea/W81/2005(H5N2). The sM2HA2CTA1/PC NPs showed protection against several lethal influenza sub-types, and the protection remained for up to 6 months following the vaccination. Therefore, sM2HA2CTA1/PC NPs may be an attractive option for a global influenza vaccine (Chowdhury et al., 2017).

Another study investigated the intranasal administration of NPs consisting of CH and poly-γ-glutamic acid (γ-PGA), and loaded with rHA antigen or an inactivated virus, for the induction of a high-degree of protective mucosal immunity in the respiratory tract (Moon et al., 2012). These researchers found that intranasal immunization with γ-PGA/CHNPs (PC NPs), containing either inactivated virus or rHA antigen, resulted in a good IgG response in the serum and an IgA response in the lung, with anti-HA neutralizing antibodies and influenza virus-specific cell-mediated immune responses. Therefore, PC NPs were capable of functioning as a potent mucosal adjuvant in comparison to cholera toxin (CT), which is a frequently used mucosal adjuvant. The mice were protected against challenge with a lethal dose of pathogenic influenza A H5N1 virus after intra-nasal administration of PC NPs loaded with rHA antigen, or the inactivated virus. It could be possible to combine PC NPs with a recombinant sub-unit influenza antigen manufactured on a large scale by a prokaryotic expression system (Moon et al., 2012).

However, low uptake of antigens by the gut-associated lymphoid tissue (GALT) leads to decreased effectiveness. Therefore a large dosage of antigen may be needed for effective immunity (Gupta et al., 2006). Mishra and colleagues (Mishra et al., 2014) tested hybrid NPs composed of CH plus *Lotus tetragonolobus* lectin (LTA) for mucosal immunization against the hepatitis B virus (HBV). In this study, the mucosal immunity was evaluated by examining salivary, intestinal, and vaginal secretions of IgA. LTA-conjugated CH-NPs evoked robust humoral and cellular responses, and could be used for oral mucosal immunization against HBV (Mishra et al., 2014).

In another study, Zhao et al. (2017) investigated NPs prepared from N-2-hydroxy-propyl trimethyl ammonium chloride modified CH (N-2-HACC) as well as N,O-carboxymethyl-modified CH (CMC) as carriers and adjuvants for vaccination. The modified CH NPs showed a higher stability and lower cytotoxicity than unmodified CH NPs. Both vehicles allowed steady release of antigens, following an early burst release. Moreover, in another study, N-2-HACC-CMC/NDV NPs administered intranasally, produced higher titers of IgA and IgG antibodies, and higher proliferation of lymphocytes, as well as increased levels of IL-2, IL-4 and IFN-γ. N-2-HACC-CMC may be a suitable delivery carrier and adjuvant for mucosal vaccines, as well as for transmucosal drug delivery (Zhao et al., 2017).

## The Effect of CH and Its Derivatives on Viral Infections

CH is widely used in both human and veterinary medicine (Muzzarelli et al., 1998), therefore, its pharmacological properties, and especially its effect on the immune system have been studied in detail. Chitosan strongly modulates the functional activity of many immune cells, such as granulocytes and macrophages. After subcutaneous implantation, CH triggered chemotaxis of *Canis familiaris* L. macrophages, and increased nitric oxide production by macrophages *in vitro*. It also stimulated leukocytosis in the peripheral blood of laboratory dogs. The secretion of nitric oxide was mainly due to the N-acetylglucosamine residues in chitosan, which was more effective than N-acetylmannosamine or N-acetylgalactosamine (Chirkov, 2002).

The function of macrophages is critical for an effective immune system response. Macrophages are antigen-presenting cells, and their interaction with T-helper cells stimulates cellular and humoral immune responses. Stimulation of macrophage functional activity with CH can also be important for suppressing viral infection in animals. In this regard, it should be mentioned that when mouse alveolar macrophages took up chitosan or chitin NPs by phagocytosis, the generation of reactive oxygen species was increased. Likewise, mouse splenocytes secreted higher amounts of γ-interferon. Interferon is known to suppress virus replication by impairing the translation ability of genomic RNAs or early viral mRNAs (Shibata et al., 1997; Chirkov, 2002). Thus, the ability of CH to stimulate interferon synthesis by macrophages could be an additional antiviral mechanism. Chitosan inhibited the reproduction of *Chlamydia trachomatis* (an obligate intracellular parasite) within HeLa cells, mainly by suppressing the uptake of parasites by neighboring cells (Pawar et al., 2008). Chitosan enhanced antiviral immune responses by increasing the production of IgG and IgA antibodies against influenza A (Texas H1N1) and influenza B (Panama) viruses (Chirkov, 2002).

Sulfated chitosan derivatives have been synthesized to specifically inhibit retrovirus replication. Studies have shown that N-carboxymethyl chitosan-N,O-sulfate could inhibit the synthesis of virus-specific proteins, and decrease HIV-1 replication in cultured T-cells, as well as the replication of Rausher murine leukemia virus in cultured mouse fibroblasts. It was found that sulfated N-carboxymethyl chitosan prevented the interaction of the viral glycoprotein gp120 (the key viral coat protein) with its receptors on T-lymphocytes. This activity lessened the entry of the virus into CD4+ cells. In addition, the modified CH competitively inhibited the activity of virus specific reverse transcriptase, most probably through interference with the enzyme binding to its polyA-oligo-dT template. The chitosan derivative showed virtually no cytotoxicity towards these cell cultures (Chirkov, 2002; Kim, 2013). The highest activity was found with a chitosan derivative sulfated at the O2 and/or O3 positions within the glucosamine residues. This derivative was effective in inhibiting the replication of HIV-1 in MT-4 lymphocytes. The anionic polymer probably bound to the positively charged V3-loop in the gp120 molecule *via* an electrostatic interaction, preventing the fusion of the virus and the cell membrane (Chirkov, 2002). It was noted that, although the ability of anionic chitosan derivatives to inhibit retroviral infection was overall similar to the effects of sulfated polysaccharides, such as heparin, dextran sulfate, etc., the effectiveness and specificity depended on the position of the sulfate groups in the glucosamine residue.

Amino groups (NH2) and hydroxyl groups (OH) are functional groups that mediate the biological activity of CH (Chen and Zhao, 2012). The molecular weight and degree of deacetylation of CH also play an important role in defining the biological activity (Tong and Chen, 2013). The number of amino groups affects the antibacterial and antioxidant activity (Klaykruayat et al., 2010; Xiao et al., 2011). Je and Kim (Je and Kim, 2006) found that aminoethyl modified chitosan provided an antioxidant effect against hydroxyl and superoxide anion radicals. Moreover, the antibacterial effects of aminoethyl-modified chitosan were seen in the inhibition of *Escherichia coli* (Meng et al., 2012). Chitosan also showed antiviral effects against viral infections in plants and phage infections in bacteria (Pospieszny et al., 1991; Chirkov, 2002; Kulikov et al., 2006). One study suggested that aminoethyl-modified CH exerted its antiviral activity by stimulation of the immune response (He et al., 2019).

Topical microbicides have been developed to prevent infection by human papillomaviruses (HPV). The main goal of these microbicides is to block the interaction between HPV virions and heparan sulfate proteoglycan (HSPG) molecules that act as receptors on the surface of the host cells (Shafti-Keramat et al., 2003). Heparin, as well as some heparin-like polysaccharides including dextran sulfate and carrageenan, have shown the ability to prevent HPV binding to the cell surface (Lembo et al., 2008). Various types of CH have also demonstrated this property (Wang et al., 2012). Sulfated CH shows a range of biological activities, including antitumor, antioxidant, anticoagulant and antiviral properties (Kulikov et al., 2006; Artan et al., 2010; Yang et al., 2013). Sulfated chito-oligosaccharide exerts an inhibitory effect on viral entry, as well as preventing virus-cell fusion through the inhibition of HIV-1gp120 binding to the CD4+ cell surface receptor. (Wang et al., 2012). Therefore, CH and its sulfated derivatives could be useful as new antiviral agents (Wang et al., 2012). To further investigate the antiviral properties of sulfated chitosans, a study by Gao et al. (2018) investigated the anti-HPV effects of 3,6-O-sulfated CH (36S). They suggested that the anti-HPV effect of 3,6-O-sulfated CH could be due to targeting the viral capsid protein, and also to regulation of the host PI3K/Akt/mTOR pathways (Gao et al., 2018).

**Figure 3:**
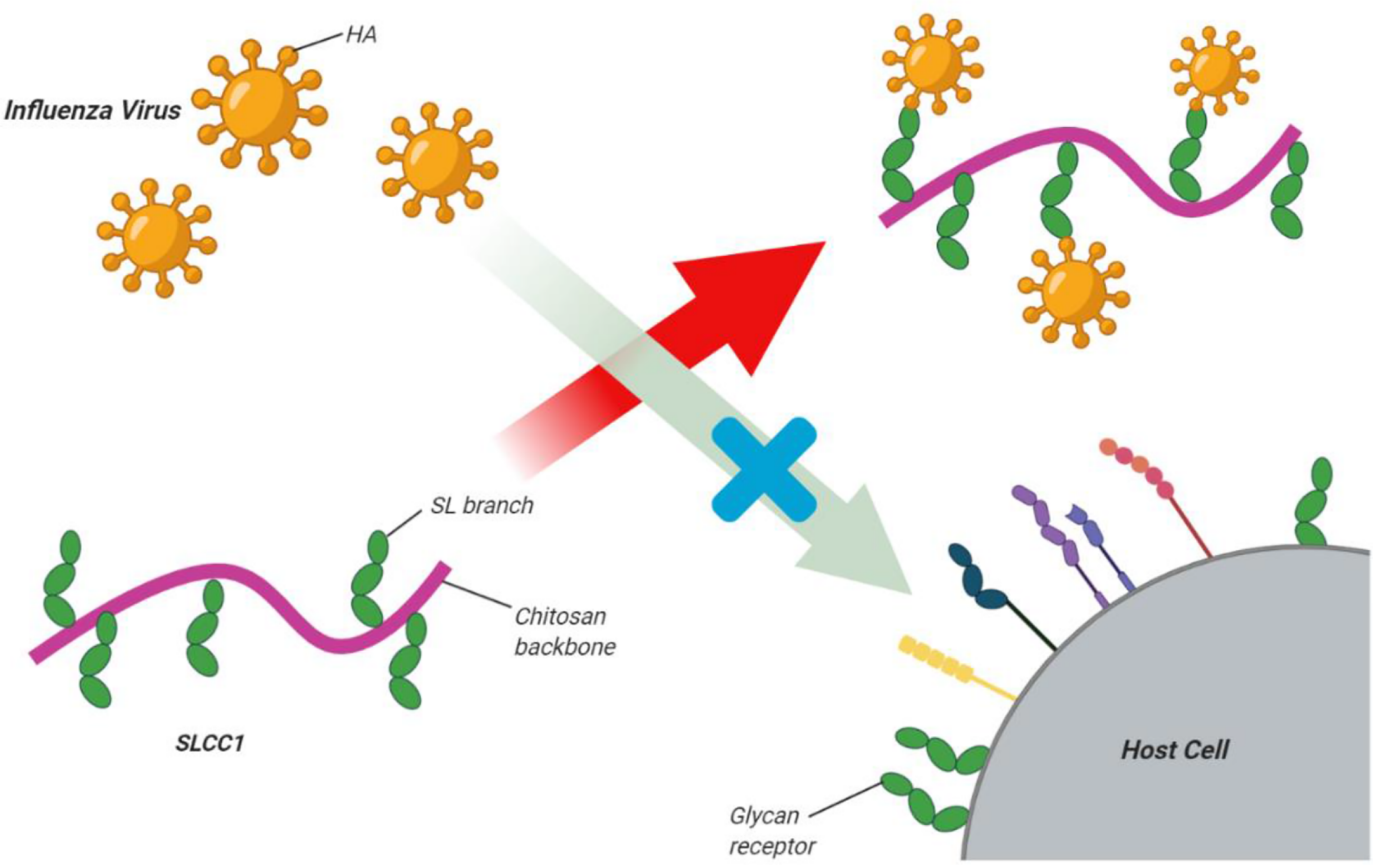
Sialyl lactose-CH (SLCC1) derivative for inhibition of influenza virus. Li et al. synthesized a sialyl lactose-CH derivative *via* graftinga lactoside aldehyde-functionalized aglycone onto the CH amino groups followed by enzymatic sialylation using sialyl-transferase. The glycosylated CH bound the influenza virus surface hemagglutinin protein with high affinityto suppress virus binding to host cells. This figure adapted from (Li et al., 2011).

Recently, it has been reported that the wide use of current anti-influenza drugs has led to the emergence of drug-resistant viruses (De Jong et al., 2005; Yen et al., 2013). Therefore, it is very important to discover new treatment approaches. Zheng and colleagues (Zheng et al., 2016) showed that the innate immune system could be stimulated by nasal administration of chitosan, and this was effective enough to protect BALB/c mice from infection with the H7N9 virus, which is also highly pathogenic to humans (Zheng et al., 2016). Su et al. (2009) reported that high-molecular-weight chitosan at high concentrations resulted in the inactivation of murine norovirus MNV-1. Li et al. synthesized a sialyl lactose-CH derivative by grafting a lactoside aldehyde-functionalized aglycone onto the CH amino groups, followed by enzymatic sialylation using sialyl-transferase. The glycosylated CH bound the influenza virus surface hemagglutinin protein with high affinity to suppress virus binding to host cells (**Figure 3**) (Li et al., 2011)

## Chitosan Vehicles for Antiviral Drug Delivery

**Figure 4:**
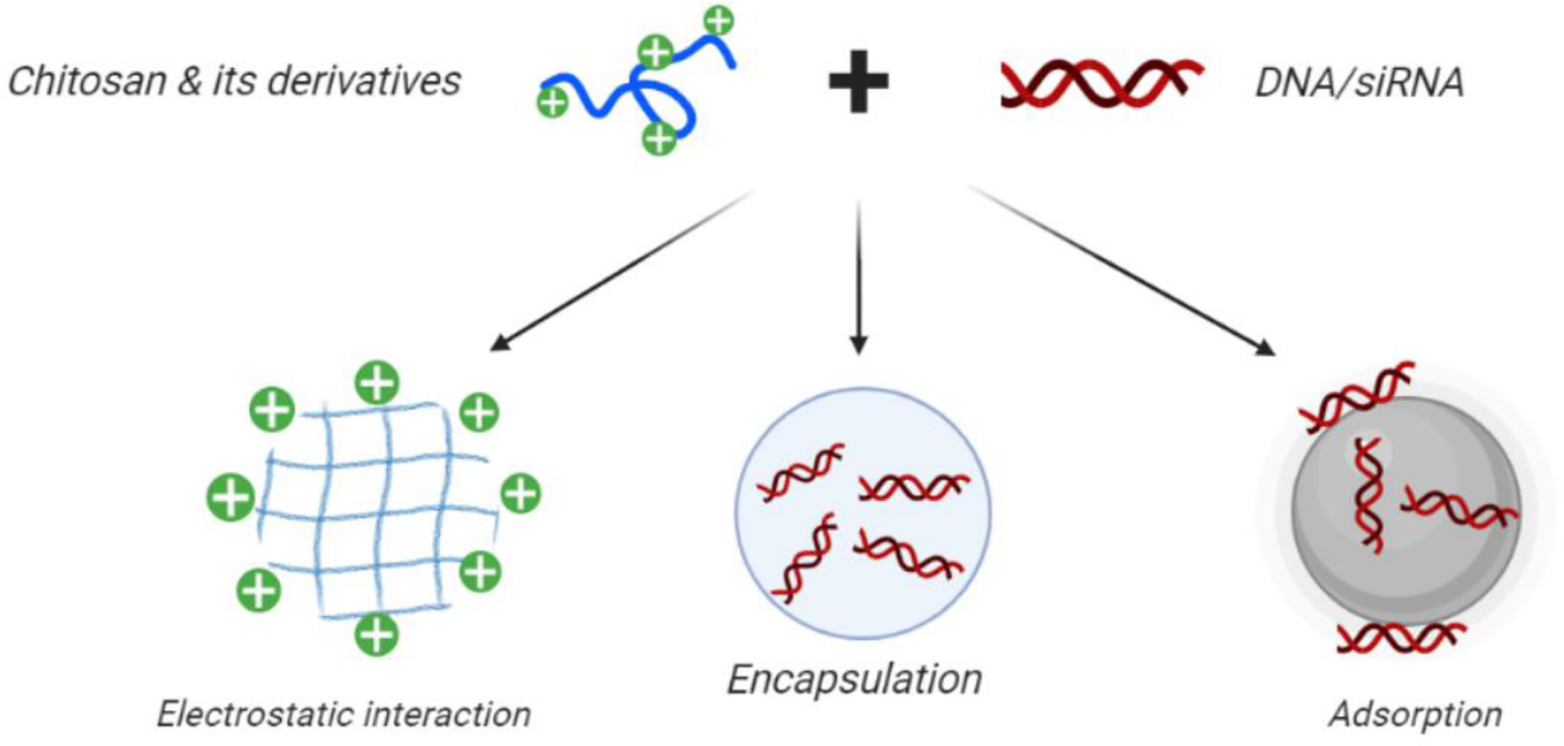
Use of CH in gene therapy and gene silencing. Chitosan nanoparticles can be loaded with plasmid DNA, antisense oligonucleotides, or small interfering RNAs for targeted gene silencing. Positively charged CH can readily form polyelectrolyte complexes with negatively charged nucleic acids. This figure adapted from (Santos-Carballal et al., 2018).

Drug delivery systems (DDS) are used for controlled and sustained drug release as well as targeted delivery to specific cells and tissues (**Figure 4**). The primary advantage of these controlled-release systems is to lessen the side effects of drugs by widening the therapeutic range between the lowest effective and toxic concentrations. This approach can enhance patient satisfaction by decreasing the number of injections, reducing the overall dose, and increasing the specificity for the diseased site. Nonetheless, DDS still suffer from some limitations, like poor biocompatibility as well as possible toxicity of the matrix itself, or its bio-degradation products. The administration route may be problematic, for example surgical operation may be needed for implantation, and their cost may be high in comparison with conventional formulations (Bernkop-Schnürch and Dünnhaupt, 2012; Hu et al., 2013).

CH has biological properties such as being hemostatic, bacteriostatic, anti-cholesteremic, anti-carcinogenic, as well as fungistatic (Saikia et al., 2015), These activities have made it a popular candidate in biomedical applications like DDS. Since CH is a naturally occurring biopolymer, its biocompatibility and lack of toxicity are viewed as advantages. CH has been approved by the FDA as a hemostatic dressing called HemCon™ for battlefield and civilian medical uses (Wedmore et al., 2006). Both low and high molecular weight CH will undergo metabolism as well as enzymatic degradation within the body, leading to removal by renal clearance (Funkhouser and Aronson, 2007). Notably, the degradation rate is dependent on the degree of deacetylation. CH can be dissolved in acid, but then becomes insoluble at physiologic (7.4) or basic pH. The presence of two hydroxyl groups on each structural unitas well as the primary amine groups, allows easy chemical modification, and facile physical interaction with metallic or metal oxide NPs, organic or inorganic compounds, and polymers (Luo and Wang, 2014; Abd Elgadir et al., 2015; Saikia et al., 2015). CH is preferred in the formulation of DDSs, because of its cationic nature allowing interaction with anionic drugs or compounds, its mucoadhesive properties, greater permeation into tissue, ability to allow cellular transfection of nucleic acids, and its suppression of multi-drug efflux pumps (Ngo et al., 2015).

Acquired immune deficiency syndrome (AIDS) is caused by infection with the HIV virus, and has caused the deaths of millions of people worldwide (das Neves et al., 2010; Sadri Nahand et al., 2020). A new therapeutic approach using a combination drug regimen, is called highly active anti-retroviral therapy (HAART) (das Neves et al., 2010). Five different types of antiretroviral drugs have been used to treat AIDS (Wong et al., 2010), each of which functions at different stages of the viral lifecycle (Gupta and Jain, 2010). For example, a protease inhibitor targets the viral protease that cleaves the GAG-POL precursor responsible for producing the viral enzymes that are required for viral proliferation (Tritch et al., 1991). Currently, the FDA has approved eight protease inhibitors, of which saquinavir has shown the highest activity. Nevertheless, saquinavir has been shown to be limited by its poor bioavailability (Li and Chan, 1999), that has been largely ascribed to a group of MDR1 (multidrug resistance 1) drug efflux pumps. P-gp mediates anti-HIV drug efflux because of their similarity to the natural substrates of P-gp mediated efflux systems (Kim A. E. et al., 1998; Kim R. B. et al., 1998). Rapid efflux of the drugs causes a lower intracellular concentration of the drug within the cells. Another mechanism ssuggested for the lower bioavailability of saquinavir is its metabolism in the liver and small intestine, that results in rapid clearance (Thummel et al., 1997). The poor bioavailability of saquinavir necessitates the use of higher doses that could encourage the emergence of drug resistance (Schapiro et al., 1996). The use of saquinavir can have many side effects like nausea, dizziness, arrhythmia, etc. Hence, nanotechnology-based delivery vehicles could be used to increase the potency of saquinavir. Ramana et al. (2014) investigated a chitosan-based nanodelivery strategy for saquinavir. In this study, the CH NPs were loaded with saquinavir and characterized by transmission electron microscopy (TEM) and differential scan calorimetry. They also measured the encapsulation efficacy, swelling properties, zeta potential, and dimensions of the NPs. The cellular uptake of CHNPs was assessed by confocal microscopy and flow cytometry. The anti-viral effectiveness was studied in cell culture. The researchers reported a 75% efficiency for saquinavir encapsulation, and a cell targeting efficiency > 92%. Saquinavir loaded CH carriers resulted in better control of viral proliferation compared to soluble drug alone, with two different HIV strains (NL4-3 and Indie-C1) and two different types of T-cells (CEM-CCR5 and Jurkat). This study demonstrated that better drug loading combined with increased cell targeting efficiency led to effective control of viral replication in the targeted T-cells (Ramana et al., 2014).

Herpes simplex virus type 1 (HSV-1) is an endemic highly transmissible virus, which is mainly transmitted by mouth-to-mouth contact. It is projected to infect up to 3.7 billion people under 50 years old (D’Affronte and Platia, 2020). In fact, HSV-1 primary infection usually occurs in childhood as an asymptomatic infection, but can develop into herpetic gingiva-stomatitis with recurrent bouts of oral labial lesions (*e.g*., fever blisters, cold sores, or herpes labialis). HSV-2, which is primarily transmitted through sexual contact, causes genital herpes which affected about 417,000,000 people globally in 2012 (Roizman, 2006; D’Affronte and Platia, 2020). Different anti-herpes drugs like famciclovir, valacyclovir or acyclovir have been approved, and are used for treating acute symptomatic herpetic infections (Andrews et al., 2006; Le Cleach et al., 2014). Moreover, recurrent orolabial mucocutaneous herpes will usually be treated with topical anti-viral agents to accelerate wound healing and reduce symptoms like pain, tingling, itching, and burning (Spruance and Kriesel, 2002). In addition, acyclovir (9-[(2-hydroxyethyl)methyl])-9H-guanine) is the treatment of choice for HSV infection. However, it should be administered several times a day to be effective, because of its short half-life as well as imperfect adsorption. Although acyclovir is used as a topical treatment (Zovirax), due to its hydrophilic nature and its poor solubility in both aqueous and fatty solvents, it does not penetrate well into the stratum corneum. New topical formulations of acyclovir have been designed to penetrate through the epidermis to reach the basal layer where the virus exists, at sufficient concentrations to inhibit replication (Prabhu et al., 2012). Donalisio and colleagues (Donalisio et al., 2018) investigated the effects of acyclovir-loaded CH nanospheres prepared by nanoemulsion templating for topical treatment of herpes-virus infections. The novel CH nanospheres were characterized by dynamic light scattering (DLS), TEM, and *in-vitro* drug release studies. A Franz cell was used to study the drug penetration through porcine skin *ex vivo*. Viral inhibition studies were done on Vero cells infected with HSV-2 and HSV-1 strains. The chitosan NS-loaded with acyclovir had a spherical shape about 200 nm in diameter and a 40.0 mV negative zeta-potential. The drug loading capacity was nearly 8.5% and 30% of the acyclovir was released from the nanospheres within 6 h. Ex vivo skin penetration was better than with acyclovir 5% cream. The acyclovir-NS complex exhibited more potent anti-viral activity compared to free acyclovir against HSV-1 as well as HSV-2 strains. Furthermore, the acyclovir-loaded NS did not display any anti-proliferative activity or any sign of cytotoxicity against host cells (Donalisio et al., 2018).

In another study, Calderón et al. (2013) prepared microparticles (MPs) as well as NPs composed of CH cross-linked with tripolyphosphate to deliver acyclovir. The system showed biocompatibility, bioadhesive properties, and potential as a penetration enhancer through the skin. The amounts of acyclovir diffused within 24 hours were as follows: 30, 40, and 80 μg for ACV alone, ACV + MP solution, and ACV loaded NPs respectively. Moreover, CH-based particles caused less tissue damage and only moderate irritation as judged by the slug mucosal irritation (SMI) assay, suggesting that ACV-NPs could be used to develop an anti-viral formulation.

In another study, Hasanovic et al. (2009) created a skin delivery system for aciclovir based on CH–tripolyphosphate NPs with good chemical stability. In this study, NPs were spontaneously formed by ionotropic gelation with tripolyphosphate. Two distinct dimensions of aciclovir-loaded NPs were characterized for their zeta-potential, particle size, and polydispersity index. A standard diffusion test with the use of a Franz diffusion-cell indicated good skin permeability dependent on the size of CH NPs, and good acyclovir loading. If the chitosan content of the NPs was higher, more acyclovir penetrated through porcine skin. Differential scanning calorimetry showed a significant reduction in the average transition temperature, showing that the NPs interacted with two layers (skin and fat). In addition, the chemical stability of acyclovir was considerably enhanced by NPs incorporation. After five weeks of photo-oxidation, the acyclovir content in the nanoparticles was remarkably higher than with a pure aqueous solution. The present study demonstrated that the incorporation of aciclovir within the CH-tripolyphosphate NPs substantially enhanced the chemical stability. Skin diffusion was better with acyclovir CHNPs, particularly those with a high CH content (Hasanovic et al., 2009).

Applications of CH for drug delivery have been limited due to its low tissue penetration as well as its poor solubility at pH> 5.6. N,N,N-trimethyl CH was prepared *via* amine functionalization, and showed improved chemical stability, solubility, biological adsorption, porosity, and non-antigenic properties (Hagenaars et al., 2009a). Therefore N-TMC has been proposed as a DDS for vaccines (**Figure 4**), drugs, biomolecules, antimicrobials, and as a scaffold matrix for skin, bone and nerve regeneration (Balmayor et al., 2012; de Britto et al., 2012; Radhakumary et al., 2011; Tsai et al., 2011).

In another study, Dehghan and colleagues (2014) investigated chitosan nanospheres to deliver an influenza vaccine. In this study, whole influenza virus combined either with CpG oligo-deoxynucleotide (CpG ODN) or with *Quillaja saponin* (QS) as adjuvants, and incorporated into CH nanospheres. Three doses of the dry powder nanosphere vaccine were administered nasally into rabbits on days 0, 45, and 60, with an additional booster dose on day 75. The cellular and humoral immune responses were examined, showing an increased titer of hemagglutination inhibition (HI) antibody in both groups in comparison with controls. CH nanospheres (WV+CpG) and CH (WV+QS) were more effective that virus alone, and CpG alone (p<0.001). The CH(WV+CpG) group showed the maximum response with rabbit serum IgG titer. The CH (WV+CpG) resulted in higher induction of sIgA titers compared to CH (WV+QS) (p<0.001), and the CpG adjuvant had a major contribution to the secretion and stimulation of IL-2 and IFN-g cytokines (3-fold & 3.5-fold increase). The CH (WV+CpG) vaccine induced the best cellular and humoral immune responses against the influenza virus following nasal administration (Dehghan et al., 2014).

CH and its derivatives have been used for organ-specific targeted drug delivery. For instance some systems can target the liver by relying on passive accumulation of DDS by the reticulo-endothelial system (RES), or else by active targeting of the liver by recognition of the ligand-modified DDS by specific hepatic receptors. One study reported the synthesis of lactosaminated N-succinyl-CH, and investigated its potential as a liver-specific drug carrier (Kato et al., 2001). This carrier bound to the asialoglycoprotein receptor (ASGP-R) and accumulated in the liver. The lactose-conjugated CH modified with PEG formed polyionic complex micelles to deliver the anti-inflammatory drug called diammonium glycyrrhizinate to treat acute HBV infection (Yang et al., 2009). Another study from Lin et al. (2008) described the modification of CHNPs by conjugation to glycyrrhizin. Conjugation was achieved by the oxidation of glycyrrhizin using sodium periodate to form aldehyde groups, that could then react with CH amino groups (Lin et al., 2008). *In-vitro* investigations showed the localization of the glycyrrhizin conjugated CH NPs in hepatocytes, with an uptake 4.9 times greater than that of non-hepatic parenchymal cells. The dose of NPs and the incubation time governed the cellular uptake, that was mediated by ligand-receptor interactions (Singh et al., 2018).

Another group used a double emulsification procedure to prepare cationic PLGA-CH NPs with HBsAg passively absorbed onto the surface, for site-specific delivery of IFN-α to hepatocytes. The HbsAg-coated (99m)Tc labeled PLGA-CHNPs showed remarkable recovery of liver function compared with unmodified PLGA NPs (Giri et al., 2011). The size of the NPs as well the hydrophobicity affected the cell-mediated and mucosal immune responses. The HBsAg-modified NPs could be administered *via* pulmonary delivery. Hydrophobic particles > 500 nm gave the best improvement in secreted IgA, IFN-γ, and interleukin-2 levels compared to hydrophilic particles < 500 nm. Larger hydrophobic particles were taken up into rat alveolar macrophages to increase the immune response (Thomas et al., 2011). Polymeric NPs formulated for HBV gene silencing using the biodegradable polymer PLGA were more effective when the cationic polymer CH was incorporated into the matrix. The plasmid DNA loading efficiency and the cellular internalization were improved. In this regard, Zeng et al. (2011) showed improved HBV silencing with CH–PLGA system, than the plain plasmid DNA (pDNA) or simple PLGA NPs. The CH–PLGA system did not show any adverse effects (Zeng et al., 2011). **Table 1** lists various chitosan nanoparticles that have been used as drug delivery systems for viral infections.

**Table 1:** Chitosan nanoparticles used as drug delivery systems for viral infections.

Chitosan NP composition	Size	Target virus	Model (*In vitro*/*In vivo*/Human)	Type of cell line	Findings	Ref
CH/AL-BV	434.6 ± 22.1 nm	Porcine reproductive & respiratory syndrome virus (PRRSV)	*In vivo*		Increased level of PRRSV-specific IgG as well as viral neutralizing antibody. Less severe interstitial pneumonia signs were reported on the microscopic evaluation and lung gross examination. Decreased viral burden in serum and tissue (lung and bronchial lymph nodes)	(Lee et al., 2018)
C-IV	222.1 nm	Viral hemorrhagic septicemia virus (VHSV)	*In vitro*		Increased anti-VHSV antibody titer following vaccination. Increased immune gene transcripts (IgT, IgM, MHC-I, pIgR, IFN-γ, MHC-II, & Caspase3).	(Kole et al., 2019)
CNPs	571.7 nm	Swine influenza A virus (SwIAV)	*In vitro* and *in vivo*	MDCK	Reduced nasal viral shedding and lung virus titers. Induced the secretion of cytokines, increased IAV-specific mucosal antibody response, and systemic specific cell mediated immune response against IAV.	(Dhakal et al., 2018)
CS/TPPr-VP28	50-80 nm	White spot syndrome virus	*In vivo*		Induced expression of immune-related genes	(Taju et al., 2018)
siRNA/CN	278 nm	Influenza virus	*In vitro* and *in vivo*	Vero	Inhibition of influenza virus replication, antiviral effects.	(Jamali et al., 2018)
CN - CpG	65–250 nm	Influenza	*In vivo*		Stimulation of humoral and cellular immunity, increased weight gain, and decreased mouse mortality.	(Sadati et al., 2018)
CN/CMD/PEI	100-150nm	HIV	*In vitro*	RAW264.7 and HEK293	Improved siRNA delivery with good cell viability. Reduced the RNA and protein expression of HIV-1 tat peptide	(Iranpur Mobarakeh et al., 2019)
CH-NP	295 ± 26 nm	HBV	*In vitro*	M cells	Elicited strong humoral and cellular immune responses.	(Mishra et al., 2014)
pFNDV-CS	199.5 nm	Newcastle virus	*In vitro*	293T	Improved immune response and prolonged release of plasmid DNA	(Zhao et al., 2014a)
CN- S	211 ± 11.50nm	HIV	*In vitro*	Jurkat cells	Better drug loading Increased cell targeting efficiency. Effective control of viral proliferation and rapid growth of targeted T cells.	(Ramana et al., 2014)
CS-TGA	240 - 252 nm	HIV	*In vitro*	VK2/E6E7 &End1/E6E7	Prevention of HIV transmission Superior biophysical properties Maximized RT of a topical microbicide.	(Meng et al., 2014)
CS-M	130-500 nm	Foot and mouth disease virus	*In vitro* and *in vivo*	BHK-21	Improved immunological parameters. Enhanced protection.	(Nanda et al., 2014)
Nac-6-IOPs	NA	Coxsackie-adenovirus	*In vitro* and *in vivo*	K562	High transduction efficiency in cells and organs. Binds to K562 cells	(Bhattarai et al., 2008)
O-2’-HACC		Newcastle virus	*In vivo*		Induced strong cellular, humoral and mucosal immune responses	(Dai et al., 2015)
N-2-HFCC/CMC	252.2 ± 32.68nm	Newcastle virus	*In vivo*		Increased lymphocyte proliferation and serum antibody titer. Increased IFN-γ and IL-2.	(Jin et al., 2017)
c-ChonS nanoPECs	NA	HIV-1	*In vitro*	PBMCs	Non-cytotoxic to PBMCs Dose-dependent decrease ofHIV-1 infection.	(Wu et al., 2016)
CH-PLGA-DNA and CH-Tre-Inactivated	500 nm	Type A foot-and-mouth disease	*In vivo*		Increased mucosal, systemic, and cell-mediated immunity.	(Pan et al., 2014)
f-TFV CS NPs-Gel	545.1 ± 69.17nm	HIV virus	*In vitro*	L929	Controlled release of tenofovir. Prevented HIV transmission.	(Timur et al., 2018)
CS-PCL	125.64 ± 6.51 nm	Influenza A virus (A/California/07/2009) H1N1	*In vivo*		Mucosal & systemic humoral& cellular immune response.	(Gupta et al., 2011)
CS/TPP-HA	358.67 ± 5.13nm	Hemagglutinin (HA)-split influenza virus	*In vivo*		Increased number of IFN-γ-secreting cells in spleen. Markedly reduced influenza morbidity.	(Sawaengsak et al., 2014)
N-TMC NPs	66 ± 13, 76 ± 9 nm	Hepatitis B virus	*In vivo*		Treated hepatitis B & allergic rhinitis.	(Subbiah et al., 2012)
C-RGVP	200-400 nm	Hepatitis B virus	*In vivo*		–	(Kim and Kang, 2008)
HBsAg CH NP	00-250 nm	Hepatitis B surface antigen	*In vivo*		Increased anti-HB secreting cells. Generated robust durable immunity. Increased Tfh cells (BAFF-R (+) B-cells and CD138+plasma cells).	(Lugade et al., 2013)
mPEG-PLA-PEI	88.9nm	Hepatitis B virus	*In vitro*	PLC/PRF/5	siRNA delivery	(Wang et al., 2010)
CH nanoemulsion	120-160 nm	Hepatitis E virus	*In vitro*	HeLa & THP1	Good cell uptake and release No cytotoxicity	(Rani et al., 2018)
pFDNA-CS/PLGA-NPs	699.1 ± 5.21 nm	Newcastle disease virus	*In vitro* and *in vivo*	293T	Controlled release of plasmid DNA. Induced strong cellular, humoral, & mucosal immune responses.	(Zhao et al., 2014b)
CH-NS/MS & HTCC-NS/MS	60 nm	Human corona viruses, HCoV-NL63 &HCoV-OC43 Mouse hepatitis virus (MHV)	*In vitro*	LLC-MK2, HCT 8 & LR7	Genipin used to cross-link CH Could be used for concentration of virus from samples or purification of water supply	(Ciejka et al., 2017)
PLGA-CHS NS	60 nm	Hepatitis B virus	*In vitro*	HepG2.2.15	Able to treat viral hepatitis in-vivo.	(Zeng et al., 2011)
CTS-Fe_3_O_4_ NPs	NA	HCV	*In vivo*		Increased antibody production and T-cell activity.	(Yang et al., 2011)
NDV/La Sota-N-2-HACC-NPs	303.88 ± 49.8nm	Newcastle disease viruses	*In-vitro* & *in-vivo*	293-T	Increased strong humoral, mucosal and cellular immune response.	(Zhao et al., 2016)
C- QCH4-dsRNA	400nm	Yellow head virus	*In vitro*	Sf9	Effective dsRNA carrier	(Theerawanitchpan et al., 2012)
CH-O-isopropyl-5’-O-d4T monophosphate	166.8nm	HIV	*In vitro*	MT4	Prevented leakage of cargo from the NPs before reaching targeted viral reservoir.	(Yang et al., 2010)
D- CN	382 ± 18nm	HIV	*In vivo*		Improved delivery system	(Al-Ghananeem et al., 2010)

## Chitosan Vehicles for Antiviral Vaccination

Influenza occurs in pigs (swine flu) caused by the influenza A-virus (IAV) of the Orthomyxoviridae family. Influenza is an economically significant disease in the pig farming industry (Dykhuis-Haden et al., 2012; Crisci et al., 2013). Swine IAV (SwIAV) infection causes an acute febrile respiratory illness, which is frequently followed by secondary bacterial infections (Dykhuis-Haden et al., 2012). Moreover, the SwIAV virus modifies its genetic diversity *via* repeated antigenic drifts or shifts. The main circulating strains of SwIAV in pigs are H1N1, H1N2, and H3N2 (Vincent et al., 2008). Since pig respiratory tract epithelial cells have receptors for human IAVs as well as avian IAVs, pigs may become infected with IAV strains from various hosts. SwIAV infections allow genetic recombination as well as adaptation of new influenza strains that may infect humans and animals, and even cause epidemics (Ito et al., 1998). Commercially available swine flu vaccines are based on multivalent whole inactivated virus (WIV) vaccines given intramuscularly (IM). (Vincent et al., 2017). These WIV vaccines protect against homologous virus infection, however they cannot induce sufficient heterologous immunity against the continually evolving IAVs caused by point mutations (Van Reeth and Ma, 2012; Vincent et al., 2017).

In addition, when the IM pathway is utilized for WIV vaccines, it will not produce an effective mucosal immune response, that is really necessary to provide cross-protective immunity against different types of IAV (Tamura and Kurata, 2004; van Riet et al., 2012). An intranasal (IN) vaccine, which targets the respiratory tract mucosal immune system, could be an improvement over IM influenza vaccines employed in pigs. Additionally, nasal mucosal vaccination results in the induction of a powerful protective immune response in the respiratory tract, and also improves immunity at the distal mucosal and systemic sites (Neutra and Kozlowski, 2006; Kim and Jang, 2017). Various kinds of NPs have been studied for IN delivery of antigens in influenza vaccines. For instance, IN immunization in a mouse model using liposome-based DNA and influenza nanovaccines induced cellular, mucosal and humoral immune responses (Wang et al., 2004). Furthermore, poly (lactic-co-glycolic) acid (PLGA) NPs loaded with highly-conserved H1N1 influenza virus peptides and administered IN triggered epitope-specific T-cell responses, as well as protective immunity in pigs (Hiremath et al., 2016). A ferritin-based IN influenza nanovaccine increased mucosal IgA secretion, T-cell responses and provided homo-subtype and hetero-subtype protection in mice (Qi et al., 2018).

Hepatitis virus B (HBV) is a severe chronic infectious liver disease that can cause cirrhosis, hepatocellular carcinoma, and even mortality if left untreated. It is estimated there are 4.7 million clinical cases of acute HBV annually (Beisel et al., 2020). Considering that effective drug treatments for HBV are lacking, parenteral vaccines involving recombinant DNA encoding HBsAg are used to protect nearly 95% of the recipients (Kwon and Lee, 2011). However the need for injection is expensive and causes patient dissatisfaction. Thus, a different administration route like intranasal (i.n.) using an appropriate DDS may be an alternative (Illum, 2003). The nasal mucosa has plentiful nasal-associated lymphoid tissue (NALT), a larger surface area, the presence of dendritic cells, as well as less enzymatic degradation compared to the oral route (Kang et al., 2009; Slütter et al., 2010). Finally, nasally administered drugs may have higher concentration, no first-pass effects, better permeation, and more patient compliance, without any side effects (Al-Qadi et al., 2011; Taranejoo et al., 2011).

Antigens encapsulated inside NPs and administered intranasally showed a higher uptake, a more controlled release of the antigens that can pass through the nasal membranes to reach the vasculature with higher immunogenicity and a systemic immune response (Kyd et al., 2001; Costantino et al., 2007). Moreover, DDS greater than 100 nm in size administered intranasally have a long residence time and mucoadhesive properties according to their surface charge, preventing polymer bio-degradation (Slütter et al., 2010).

Hepatitis-B virus surface antigen (HBsAg) was loaded into N,N,N-trimethyl chitosan NPs (N-TMC NPs) for controlled intra-nasal delivery as reported by Subbiah et al. (2012). The NP size was tunable by modifying the N-TMC content, giving 66 ± 13 nm for 0.25 wt%, and 76 ± 9 nm for 0.5 wt.%. A HBsAg loading of 380 and 760 µL/mL resulted in 143 ± 33, 259 ± 47 nm-sized spherical N-TMC NPs with a loading efficiency of HBsAg-antigen of 90%-93% and 96%-97% respectively. I*n-vivo* immunological studies were conducted on 6-8 week old female BALB mice over 43 days, showing the high stability and efficiency of this HBV vaccination (Subbiah et al., 2012).

Recent approaches to treat hepatitis B have attempted to clear active HBV infections by suppressing viral replication. Some nucleoside or nucleotide analogs have been used as anti-viral drugs, to efficiently suppress HBV replication. In addition, lamivudine (LA) targets the host cell nucleus and inhibits the reverse transcriptase enzyme in order to terminate HBV replication (Asselah et al., 2005). There are some limitations of anti-viral drugs, such as non-specific biodistribution in-vivo, enzymatic metabolism, and issues in transportation across biological membranes (Langer, 1998). These limitations reduce the therapeutic benefit, require larger doses, and lead to side effects well as drug resistance. Moreover, by utilizing pharmaceutical engineering, the delivery of anti-viral drugs to their molecular targets will increase efficiency and decrease adverse effects. Earlier investigations revealed that the molecular activity of anti-HBV drugs largely takes place in subcellular organelles of the host cells, in addition to inhibiting HBV binding to its receptors (Zoulim and Perrillo, 2008). Thus, nanotechnology-based vehicles that can target sub-cellular organelles are an example of molecular targeted therapy (Torchilin, 2008). One example is stearic acid-grafted CH oligosaccharide polymeric micelles, that could target specific sub-cellular organelles (You et al., 2007; You et al., 2008).

Hydrophobic drugs are loaded into the hydrophobic core of the micelles *via* hydrophobic interactions, and possibly by metal-ligand coordination bonds (Nishiyama et al., 2001) or electrostatic interactions (Kabanov et al., 1996). However, lamivudine (LA) has only very low hydrophobicity, which can be increased by chemical modification. For instance, lamivudine stearate (LAS) was synthesized by esterification of LA with stearic acid by Li et al. (2010). After esterification, the octanol:water distribution coefficient (log P) of LA was increased from -0.95 to 1.82. Moreover, g-CH oligosaccharide modified with stearic acid (CSO-SA) formed micelles that could be loaded with LAS showing rapid internalization and accumulation in tumor cells. CSO-SA with a 3.79% amino substitution degree (SD) showed a critical micellar concentration (CMC) of 0.032 mg/ml, and the micelles of a 1 mg/ml CSO-SA concentration showed a mean diameter of 460.8 nm with a narrow size distribution and zeta potential of +29.7 mV. After LAS loading, the micelle size decreased and the zeta-potential increased. The LAS-loaded CSO-SA micelles (CSO-SA/LAS) showed a pH dependent release of LA, with a greater release rate at pH 7.4 compared to pH 6.2. CSO-SA/LAS demonstrated lower cytotoxicity and higher cellular uptake of LA by HBV transfected tumor cells (HepG2.2.15) compared to free LA. The antiHBV activity of CSO-SA/LAS *in vitro* showed higher inhibition of antigen expression and DNA replication, in comparison to LAS and LA alone (Li et al., 2010).

Dhakal et al. (2018) assessed the immune response and cross-protective efficiency of inactivated SwIAV vaccine encapsulated in chitosan NPs and delivered *via* the IN route to pigs. Killed or inactivated SwIAV H1N2 (δ-lineage) virus antigens (KAg) were encapsulated in the CH NPs to form CNPs-KAg which was administered twice as an IN mist to nursery pigs. Vaccinated and control animals were challenged with a SwIAV H1N1 (γ-lineage). The pigs vaccinated with the CNPs-KAg showed elevated serum IgG antibodies and more mucosal IgA secretion in the nasal swabs, broncho-alveolar lavage (BAL) fluids as well as lung lysates. Protection was achieved against homologous (H1N2), heterologous (H1N1), and hetero-subtype (H3N2) influenza A-virus strains. Before the challenge, they found higher levels of cytotoxic-T lymphocyte proliferation, antigen specific lymphocyte proliferation, and IFN-γ secretion from peripheral blood mononuclear cells, in pigs immunized with CNPs-KAg compared to controls vaccinated with KAg alone. When CNP-KAg vaccinated pigs were challenged with the heterologous virus, the microscopic and macroscopic pulmonary lesions were reduced. Notably, titers of SwIAV in nasal swabs and BAL fluid were substantially reduced in pigs vaccinated with CNPs-KAg, but not in the KAg control group. Also, an increased frequency of helper T memory cells and an increase in IFNγ secretion in bronchial lymph nodes were observed. The SwIAV CH nanovaccine delivered by the IN route triggered IgA and cross-protective mucosal cellular immune responses in the respiratory tract, leading to lower virus titers in the nose and lungs. They suggested that the chitosan-based nanovaccine for influenza might be useful for commercial pig farms, and could be tested for human influenza vaccination (Dhakal et al., 2018).

**Table 2** lists some chitosan nanoparticles that have been used for improving vaccination against viral infections.

**Table 2:** Chitosan nanoparticles investigated for vaccination against viral infections.

Chitosan NP composition	Size	Target virus	Model (*In vitro*/*In vivo*/Human)	Type of cell line	Findings	Ref
GLPC	431.21 ± 0.90 nm	Respiratory system virus	*In vivo*		Antibacterial activity Antioxidant activity Effective anti-inflammatory activity	(Singh et al., 2018b)
CN/IL-12	NA	HPV-16	*In vitro* and *in vivo*	TC-1	Combined vaccination inhibited tumor progression compared with chitosan or IL-12 alone. Co-formulation of chitosan and IL-12 resulted in higher IFN-γ and IL-4 and decreased IL-10 generation.	(Tahamtan et al., 2018)
AIV-CN	149.5 nm	Avian influenza virus	*In vivo*		Increased% phagocytic activity and phagocytic index.	(Mohamed et al., 2018)
N-2-HACC and CMC	251.8 ± 10.2 nm and 122.4 ± 4.2 nm	Newcastle virus	*In vivo*		Induced cellular, mucosal and humoral immune responses as an adjuvant Induced higher titers of IgA and IgG antibodies. Promoted the proliferation of lymphocytes. Induced more IL-2, IL-4, and IFN-γ.	(Zhao et al., 2017)
IBV-CS	286nm	Avian infectious bronchitis virus	*In vivo*		Induced strong mucosal immune responses. Induced both humoral and cell-mediated immune responses. Switched from IgA isotype to IgG.	(Lopes et al., 2018)
HACC/CS and SCS	320.03 ± 0.84, 156.2 ± 9.29 nm	Newcastle virus	*In vivo*		Lower humoral immunity and higher cellular immunity.	(Yang et al., 2020b)
CS-TPP	NA	Infectious pancreatic necrosis virus (IPNV)	*In vitro* and *in vivo*	CHSE-214	Promoted gene transcription of Mx-1, IFN-1, IgT, CD4 and IgM. Decreased VP4 transcripts. Higher levels of circulating antibodies.	(Ahmadivand et al., 2017)
PC NPs	200nm	Enterovirus 71	*In vitro* and *in vivo*	Vero and Raw264.7	Increased virus-specific humoral immunity (IgG, IgG2a & IgG1) and cell-mediated immune response (IFN-γ & IL-4).	(Pathinayake et al., 2018)
c-poly(I:C) NPs	368 ± 1.3 nm	VHSV-G	*In vivo*		Significantly protected against VHSV.	(Kavaliauskis et al., 2016)
γ-PGA-PC NPs	NA	Influenza A virus strains	*In vivo*		Induced systemic immunity (IgG & IgA). Increased level of sM2- or HA2-specific cell mediated immune response. Provided cross-protection against lethal influenza sub-types.	(Chowdhury et al., 2017)
TMC	225.3 ± 23nm	Influenza virus	*In vivo*		Induced Th1 cellular and humoral immune responses. Increased IgG, IgA & IFN-γ.	(Dabaghian et al., 2018)
Plasmid DNA loaded-CN_S_	300–400 nm	Hepatitis B virus	*In vitro*	HeLa	Induced systemic and mucosal humoral and cellular immune responses.	(Khatri et al., 2008b)
CS-γ-PGA	265.3 ± 7.28 nm	HBV virus	*In vivo*		Long-term protection against HBV	(Wang et al., 2018)
C-PAP-phMGFP	NA	Yellow head virus	*In vivo*		Increased phagocytic activity.	(Khimmakthong et al., 2013)
PC NPs	900nm	Influenza A H5N1 virus	*In vivo*		Increased protective mucosal immunity in respiratory tract. Induced anti-HA IgA response in the lung. Induced IgG response in sera including anti-HA neutralizing antibodies.	(Moon et al., 2012)
H1N1-TMC/NP	39.4 ± 1.5 nm and 141.3 ± 1.9 nm	Influenza A (H1N1)	*In vivo*		Increased mucosal sIgA & serum IgG. Produced IL-1β, IL-6, IL-2, and IFN-γ. Stimulated macrophages and spleen lymphocytes.	(Liu et al., 2015)
GC- E2- NPs	304 nm	HIV-1	*In vivo*		Antiviral activity against HIV-1	(Ariza-Sáenz et al., 2017)
BCG-CWCs-CS-NPs	372.0 ± 11.2 nm	Dengue virus	*In vitro*	THP-1	Increased DC markers (CD86, HLA-DR & CD80). Induced production of cytokines and chemokines Increased THP-1 cellular uptake. Stimulated immature dendritic cells (iDCs).	(Hunsawong et al., 2015)
pRSC-NLDC145.gD-IL21 DNA/CHNPs	NA	Herpes simplex virus	*In vivo*		Alleviated symptoms of recurrent and primary herpes simplex virus keratitis. Targeted corneal DCs. Induced strong humoral & cellular immune responses.	(Tang et al., 2018)
pVAX(HBc)DNA- CH NPs	271.1 ± 6.5nm	Hepatitis B Virus	*In vivo*		Enhanced immunogenicity. Increased antibody production, IFN-γ secretion, antigen-specific cell lysis.	(Jiang et al., 2007)
CN_S_	200 nm	Hepatitis B virus	*In-vitro* & *In-vivo*	RAW 264.7	Delivered imiquimod& recombinant HBV surface antigen Increased proinflammatory cytokines TNF-α & IL-6. Enhanced IgG over time and specific immunological memory. Balanced cellular &humoral responses	(Vicente et al., 2013)
CH (WV+CpG)	581.1 ± 32.6nm	Influenza virus	*In vivo*		Increased hemagglutination inhibition (HI) antibody titer Increased sIgA and IgG titers Stimulated IFN-γ & IL-2 secretion	(Dehghan et al., 2014)
N,N,N-trimethyl chitosan (TMC)	200–220 nm	Influenza virus	*In vivo*		Increased IgG1, IgG2a/c and IgG responses Improved immunogenicity and protected against viral challenge.	(Hagenaars et al., 2009b)
Chitosan-pJME/GM-CSF NPs	108.3 nm	Japanese encephalitis virus	*In vivo*		Increased splenic DC activity & cell mediated immunity.	(Zhai et al., 2015)
Chitosan-JEV DNA vaccines	200 nm	Japanese encephalitis virus	*In vivo*		Activated DCs in hair follicles & epidermis.	(Huang et al., 2009)
pCAGG-ChIL2 CNPs	100 and 200 nm	Newcastle disease virus (NDV)	*In vivo*		Increased hemagglutination inhibition antibody titer& serum IFN-γ.	(Zhang et al., 2010)

## Chitosan Vehicles for Delivery of RNA-Based Therapeutics

As mentioned earlier, nucleic acid-based drugs have been introduced as a treatment for various conditions. Nucleic acid-based drugs can be divided into two main groups called double-stranded RNA-mediated interference (RNAi) and antisense oligonucleotides (ASO) (Chery, 2016). RNAi is used for post-transcriptional gene silencing, mediated by ribonucleases in combination with other complexes and enzymes that cleave the targeted messenger RNA into small segments (Agrawal et al., 2003). Antisense oligonucleotides bind to the respective target nucleic acids by Watson-Crick base pairing, which inhibits or alters gene expression through splicing modification, target degradation, steric hindrance, etc. In contrast to small molecules drugs, that work by binding to proteins with frequent off-target toxic side effects (Sharma et al., 2014), RNA-based drugs are much more specific by targeting individual nucleic acids. Nucleic acids based drugs have more specificity, lower toxicity, and higher activity. Two major diseases, spinal muscular atrophy (SMA) and Duchenne muscular dystrophy (DMD) are good examples of those conditions, which have received approval for treatment by these approaches (Kole et al., 2012). Although RNA-based drugs enjoy enormous potential, some potential challenges should be considered. For example, RNAs have been proven to be innately unstable. Hence, delivering nucleic acids in quantity to the target tissues is difficult because of nuclease-mediated degradation in the circulation and clearance by renal excretion (Moreno and Pêgo, 2014). Moreover, some other issues such as toxicity as a result of over-activation of the immune system, as well as off-targeting effects should be investigated (Davidson and McCray, 2011).

The ease of chemical modification of chitosan, as well as the possibility to tailor its structural and functional properties, are due to the plentiful hydroxyl and amino groups in the CH chains (Singh et al., 2014). The amino groups are responsible for the positive charge formed at pH < 6, that equates to the pH of tumoral extra-cellular medium. Nevertheless, the cationic charge of CH disappears at the physiological pH of the blood, resulting in lower NP stability, and poor efficiency in siRNA complex formation. Stability is a key concern for *in vivo* gene silencing applications, along with interactions with various serum proteins after systemic delivery (Ragelle et al., 2013). Nonetheless, because positively charged CH is a natural polymeric structure, it has been widely explored for the delivery of nucleic acids (Zhang et al., 2013). A key advantage is the strong electrostatic interactions with the negatively charged RNA, which creates stable polyplexes. In addition, the siRNA binding activity of CH is clearly higher than other natural polysaccharides, that commonly possess negative or neutral charges. For instance, another natural polysaccharide, hyaluronic acid required to be to be chemically modified to become a cationic polymer in order to be applied in the delivery of nucleic acids (Serrano-Sevilla et al., 2019).

In one study, Wang et al. used nano-precipitation and solvent evaporation procedures to prepare four types of cationic NPs for delivery of siRNA to target HBV virus infections. Poly(D,L-lactide-co-glycolide) (PGLA) or mPEG-PLA was attached to either PEI or CH as a surface coating, and the size and size distribution of the NPs were measured by laser scattering, surface charge by zeta potential, and surface chemistry by X-ray electron spectroscopy (XPS). The mPEG-PLA-PEI NPs showed the best siRNA transfection efficiency and the highest inhibition of the expression of HBV surface antigen (Wang et al., 2010).

Another research group created CH/siRNA NPs as a possible treatment option against influenza virus infections *in vivo* and *in vitro* (Jamali et al., 2018). They formulated a siRNA sequence against influenza nucleoprotein, incorporated with CH polymer as a siRNA/chitosan NP complex. They used dynamic light scattering to measure the NP zeta-potential and size. Fluorescence microscopy was used to visualize the uptake of the labeled siRNA into Vero-cells. Flow cytometry was used to analyze and quantify the NP-mediated knockdown of the enhanced green fluorescent protein (EGFP) gene in Vero cells. The CH/siRNA NPs were efficiently taken up by Vero cells, and inhibited the replication of influenza virus. Nasal delivery of the siRNA using the CH-NP complex showed anti-viral activity, resulting in significant protection of BALB/c mice from a lethal influenza challenge. They concluded that CH NPs equipped with siRNA had the potential to control influenza virus infections (Jamali et al., 2018).

MiRNA-based therapeutics are being investigated to restore the expression of down-regulated miRNAs, or inhibit the expression of unwanted mRNAs (Chaudhary et al., 2018). For example, McKiernan et al. explored the treatment of cystic fibrosis using a miRNA-based nanomedicine. These researchers utilized a nano-delivery system composed of cytosine and PEI for targeting of miR-126 (McKiernan and Greene, 2015). Moreover, Deng et al. described the delivery of a combination of miR-34a plus doxorubicin (DOX) to treat breast cancer employing hyaluronic acid-CH-NPs (Deng et al., 2014). Louw et al. used CH-mediated delivery of miRNA-124 to reduce the number and activation state of microglial cells in spinal cord injuries in rats (Louw et al., 2016).

Chitosan-based nanocarriers are expected to be useful for the delivery of small interfering RNAs, microRNAs, or antisense oligonucleotides, as a molecular targeted therapy in the near future.

## Conclusions

Nanomedicines are being increasingly used for drug delivery, with many advantages, including tissue targeting, controlled release, improved permeability and solubility of drugs, greater effectiveness, improved safety, and lower toxicity. Naturally occurring materials are often preferred for the construction of these nanomedicines, compared to synthetic polymers, inorganic materials, or carbon-based nanomaterials. The bio-pharmaceutical properties of these natural nanomedicines, include low toxicity, and improvements in cell uptake, biodistribution, metabolism, and excretion. The possible long-term accumulation of non-biodegradable nanoparticles in tissue and organs, has led to some concerns about possible carcinogenicity and genotoxicity. Metallic NPs (gold and silver), carbon-based NPs (carbon nanotubes and graphene), and inorganic NPs (silica and titania) have all been faced with these worries and concerns. On the other hand, there have been many naturally occurring biodegradable materials that have been used to prepare various types of nanostructures, including proteins, peptides, lipids, and polysaccharides such as cellulose. Nevertheless, we believe that chitosan is one of the most promising types of naturally occurring materials in the nanomedicine arena. This preference is based on the well-known lack of toxicity of chitosan, as illustrated by the fact that chitosan is a food ingredient and is widely consumed by human beings as a health-food supplement. Another very important property of chitosan is its mucoadhesive ability that allows CH NPs to be administered by transmucosal routes, such as intranasal, intraocular, intravaginal, intratracheal or intrapulmonary etc. This ability is highly relevant when it comes to consider viral diseases, which often gain entry to the human body *via* a mucosal route.

Viral infections are a universal challenge to the human race that affect the health and economic well-being of millions of people, and cause disability, suffering, and death throughout the world. Treatment of viral infections has been challenging because viruses hijack the host cells in order to proliferate, and killing the virus often means killing the host cell as well. Some antiviral drugs have been developed based on nucleoside analogs and inhibitors of viral-specific enzymes. However, drug resistance often emerges due to the ability of viruses to undergo relatively facile mutations. Drug resistance is considered to be a significant threat to public health, leading to an increase in mortality and medical expenses. Infectious virus particles (virions) attach to specific receptors on susceptible cells leading to their entrance, and establishment of a viral infection. Viruses can spread within the body by local invasion, or by long distance transport *via* the bloodstream, lymphatics, or neuronal pathways. Cell-to-cell transmission of infectious viruses involves the direct export of the infectious particles out of the cell into the extra-cellular environment. The virus particles can be transported along nerve cells and spread to epithelial cells.

In addition to anti-viral drugs, vaccines are an important therapeutic approach that can not only prevent the development of a viral infection, but can also decrease the severity of the infection once it has become established. Vaccines have been proved to be highly effective against many viral infections, but many vaccines are poorly effective or even completely ineffective. The reasons for this lack of universal efficacy are manifold, and some are still under investigation. For this reason many groups have studied the use of various NPs to deliver different kinds of vaccines, whether they be whole inactivated viruses, recombinant viral antigens, or DNA and RNA sequences. Moreover, the NPs themselves can act as an adjuvant, by increasing the uptake of the vaccine by antigen presenting cells. Additional adjuvants can be combined with the vaccine inside the NPs to further improve the immunogenicity.

Chitosan-based NPs have an intrinsic positive charge, which can interact with the negative charge present on call membrane and on mucosal surfaces accounting for its mucoadhesive properties. This is useful because delivering the vaccines across the mucosal surface allows the induction of specific mucosal immunity characterized by secretion of IgA antibodies as well as IgG and IgM. Moreover the mucosal-associated lymphoid tissue (MALT) can be activated by vaccines encapsulated in CH NPs and delivered to the mucosal surface, particularly by the intranasal administration route.

Chitosan-based nanocarriers show many advantageous properties, such as nanoscale dimensions, high surface area to volume ratio, as well as the ability to tailor the surface charge and attach targeting ligands. Moreover, the chitosan matrix is able to incorporate a wide variety of different types of cargo, including antiviral drugs, proteins, peptides, nucleic acids, and even whole inactivated viruses. One relatively new approach to treating viral infections is based on gene silencing. Gene silencing uses small interfering RNAs, microRNAs, or antisense oligonucleotides, all of which can be loaded into CH NPs. The idea is to recognize the 3’-untranslated region of the viral mRNA to allow it to be degraded by the RNA-induced silencing complex (RISC). Chitosan nanostructures are also able to cross biological barriers such as the blood-brain barrier (BBB). CH NPs can increase drug or gene delivery to the site of the viral infection and also improve the cellular uptake.

As mentioned earlier, CH-based carriers enjoy increasing popularity due to their many advantages. Nonetheless, they still suffer from several limitations, like poor solubility at the physiological pH, premature release in the cytoplasm, as well as questions about the stability of the complexes following cellular uptake. Therefore, it is necessary to discover further improvements in CH-based nanocarriers. Researchers have employed chemical modification of CH using PEG (PEGylation) to improve CH solubility, although excessive PEGylation can decrease the density of the positive charges on CH, and lower its ability to bind to nucleic acids. Furthermore, chemical modification made to the siRNA itself may enhance the stability of the NPs, although this approach can make the siRNA activity less efficient. The addition of other components with a negative charge has been proposed to increase the stability of the CH NPs to improve gene silencing. CH NPs often show a higher encapsulation efficiency (EE%), and the ability for sustained release after uptake into the cells. Additional experiments are warranted in animal models of diseases such as viral infections and unwanted fibrosis. However, future researchers must optimize the CH modification procedures for full realization.

## Author Contributions

HM and MH contributed in conception, design, statistical analysis and drafting of the manuscript. HB, FB, SM, ZS, JS, MN, HB, BB, MA-K, and MG contributed in data collection and manuscript drafting. All authors contributed to the article and approved the submitted version.

## Conflict of Interest

MH declares the following potential conflicts of interest. Scientific Advisory Boards: Transdermal Cap Inc, Cleveland, OH; BeWell Global Inc, Wan Chai, Hong Kong; Hologenix Inc. Santa Monica, CA; LumiTheraInc, Poulsbo, WA; Vielight, Toronto, Canada; Bright Photomedicine, Sao Paulo, Brazil; Quantum Dynamics LLC, Cambridge, MA; Global Photon Inc, Bee Cave, TX; Medical Coherence, Boston MA; NeuroThera, Newark DE; JOOVV Inc, Minneapolis-St. Paul MN; AIRx Medical, Pleasanton CA; FIR Industries, Inc. Ramsey, NJ; UVLRx Therapeutics, Oldsmar, FL; Ultralux UV Inc, Lansing MI; Illumiheal&amp;Petthera, Shoreline, WA; MB Lasertherapy, Houston, TX; ARRC LED, San Clemente, CA; Varuna Biomedical Corp. Incline Village, NV; Niraxx Light Therapeutics, Inc, Boston, MA. Consulting; Lexington Int, Boca Raton, FL; USHIO Corp, Japan; Merck KGaA, Darmstadt, Germany; Philips Electronics Nederland B.V. Eindhoven, Netherlands; Johnson &amp; Johnson Inc, Philadelphia, PA; Sanofi-Aventis Deutschland GmbH, Frankfurt am Main, Germany. Stockholdings: Global Photon Inc, Bee Cave, TX; Mitonix, Newark, DE.

The remaining authors declare that the research was conducted in the absence of any commercial or financial relationships that could be construed as a potential conflict of interest.

## References

[B1] Abd Elgadir M. Uddin Md S. Ferdosh S. Adam A. Chowdhury A. J. K. Sarker Md Z. I. (2015). Impact of chitosan composites and chitosan nanoparticle composites on various drug delivery systems: A review. J. Food Drug Anal. 23, 619–629. 10.1016/j.jfda.2014.10.008 28911477 PMC9345468

[B2] Agrawal N. Dasaradhi P. V. Mohmmed A. Malhotra P. Bhatnagar R. K. Mukherjee S. K. (2003). RNA interference: biology, mechanism, and applications. Microbiol. Mol. Biol. Rev. 67, 657–685. 10.1128/MMBR.67.4.657-685.2003 14665679 PMC309050

[B3] Ahmadivand S. Soltani M. Behdani M. Evensen Ø. Alirahimi E. Hassanzadeh R.. (2017). Oral DNA vaccines based on CS-TPP nanoparticles and alginate microparticles confer high protection against infectious pancreatic necrosis virus (IPNV) infection in trout. Dev. Comp. Immunol. 74, 178–189. 10.1016/j.dci.2017.05.004 28479343

[B4] Akagi T. Higashi M. Kaneko T. Kida T. Akashi M. (2005). In vitro enzymatic degradation of nanoparticles prepared from hydrophobically-modified poly (γ-glutamic acid). Macromol. Biosci. 5, 598–602. 10.1002/mabi.200500036 15991216

[B5] Alexis F. Pridgen E. Molnar L. K. Farokhzad O. C. (2008). Factors affecting the clearance and biodistribution of polymeric nanoparticles. Mol. Pharm. 5, 505–515. 10.1021/mp800051m 18672949 PMC2663893

[B6] Al-Ghananeem A. M. Saeed H. Florence R. Yokel R. A. Malkawi A. H. (2010). Intranasal drug delivery of didanosine-loaded chitosan nanoparticles for brain targeting; an attractive route against infections caused by AIDS viruses. J. Drug Target 18, 381–388. 10.3109/10611860903483396 20001275

[B7] Al-Qadi S. Grenha A. Remuñán-López C. (2011). Microspheres loaded with polysaccharide nanoparticles for pulmonary delivery: Preparation, structure and surface analysis. Carbohydr. Polymers 86, 25–34. 10.1016/j.carbpol.2011.03.022

[B8] Andrews W. W. Kimberlin D. F. Whitley R. Cliver S. Ramsey P. S. Deeter R. (2006). Valacyclovir therapy to reduce recurrent genital herpes in pregnant women. Am. J. Obstet. Gynecol. 194, 774–781. 10.1016/j.ajog.2005.11.051 16522412

[B9] Ansari R. Sadati S. M. Mozafari N. Ashrafi H. Azadi A. (2020). Carbohydrate Polymer-Based Nanoparticle Application in Drug Delivery for CNS-Related Disorders. Eur. Polymer J. 128, 109607.

[B10] Ariza-Sáenz M. Espina M. Bolaños N. Calpena A. C. Gomara M. J. Haro I.. (2017). Penetration of polymeric nanoparticles loaded with an HIV-1 inhibitor peptide derived from GB virus C in a vaginal mucosa model. Eur. J. Pharm. Biopharm. 120, 98–106. 10.1016/j.ejpb.2017.08.008 28842284

[B11] Artan M. Karadeniz F. Karagozlu M. Z. Kim M.-M. Kim S.-K. (2010). Anti-HIV-1 activity of low molecular weight sulfated chitooligosaccharides. Carbohydr. Res. 345, 656–662. 10.1016/j.carres.2009.12.017 20117763

[B12] Asselah T. Ripault M.-P. Castelnau C. Giuily N. Boyer N. Marcellin P. (2005). The current status of antiviral therapy of chronic hepatitis B. J. Clin. Virol. 34, S115–SS24. 16461210 10.1016/s1386-6532(05)80020-4

[B13] Badawy M. E. I. Rabea E. I. (2011). A biopolymer chitosan and its derivatives as promising antimicrobial agents against plant pathogens and their applications in crop protection. Int. J. Carbohydr. Chem. 2011.

[B14] Balmayor E. R. Baran E. T. Azevedo H. S. Reis R. L. (2012). Injectable biodegradable starch/chitosan delivery system for the sustained release of gentamicin to treat bone infections. Carbohydr. Polymers 87, 32–39. 10.1016/j.carbpol.2011.06.078 34662968

[B15] Beisel C. Addo M. M. zur Wiesch J. S. (2020). Seroconversion of HBsAG coincides with hepatitis A super-infection: A case report. World J. Clin. cases 8, 1651. 10.12998/wjcc.v8.i9.1651 32432143 PMC7211520

[B16] Bernkop-Schnürch A. Dünnhaupt S. (2012). Chitosan-based drug delivery systems. Eur. J. Pharmaceutics Biopharm. 81, 463–469. 10.1016/j.ejpb.2012.04.007 22561955

[B17] Bhattarai S. R. Kim S. Y. Jang K. Y. Lee K. C. Yi H. K. Lee D. Y.. (2008). N-hexanoyl chitosan-stabilized magnetic nanoparticles: enhancement of adenoviral-mediated gene expression both *in vitro* and *in vivo* . Nanomedicine 4, 146–154. 10.1016/j.nano.2008.02.001 18374634

[B18] Bowman M. C. Ballard T. E. Ackerson C. J. Feldheim D. L. Margolis D. M. Melander C. (2008). Inhibition of HIV fusion with multivalent gold nanoparticles. J. Am. Chem. Soc. 130, 6896–6897. 10.1021/ja710321g 18473457 PMC2916654

[B19] Bramwell V. W. Perrie Y. (2006). Particulate delivery systems for vaccines: what can we expect? J. Pharm. Pharmacol. 58, 717–728. 10.1211/jpp.58.6.0002 16734973

[B20] Brunel F. Véron L. David L. Domard A. Delair T. (2008). A novel synthesis of chitosan nanoparticles in reverse emulsion. Langmuir 24, 11370–11377. 10.1021/la801917a 18774829

[B21] Calderón L. Harris R. Cordoba-Diaz M. Elorza M. Elorza B. Lenoir J.. (2013). Nano and microparticulate chitosan-based systems for antiviral topical delivery. Eur. J. Pharm. Sci. 48, 216–222. 10.1016/j.ejps.2012.11.002 23159663

[B22] Calvo P. Remunan-Lopez C. Vila-Jato J. L. Alonso M. J. (1997). Novel hydrophilic chitosan-polyethylene oxide nanoparticles as protein carriers. J. Appl. Polymer Sci. 63, 125–132. 10.1002/(SICI)1097-4628(19970103)63:1<125::AID-APP13>3.0.CO;2-4

[B23] Caron J. Reddy L. H. Lepêtre-Mouelhi S. Wack S. Clayette P. Rogez-Kreuz C.. (2010). Squalenoyl nucleoside monophosphate nanoassemblies: new prodrug strategy for the delivery of nucleotide analogues. Bioorg. Med. Chem. Lett. 20, 2761–2764. 10.1016/j.bmcl.2010.03.070 20363623

[B24] Chaudhary V. Jangra S. Yadav N. R. (2018). Nanotechnology based approaches for detection and delivery of microRNA in healthcare and crop protection. J. Nanobiotechnol. 16, 40. 10.1186/s12951-018-0368-8 PMC5897953 29653577

[B25] ,Cheba B. A. (2011). Chitin and chitosan: marine biopolymers with unique properties and versatile applications. Global J. Biotechnol. Biochem. 6, 149–153.

[B26] Chen J. L. Zhao Y. (2012). Effect of molecular weight, acid, and plasticizer on the physicochemical and antibacterial properties of β-chitosan based films. J. Food Sci. 77, E127–EE36. 23163939 10.1111/j.1750-3841.2012.02686.x

[B27] Chery J. (2016). RNA therapeutics: RNAi and antisense mechanisms and clinical applications. Postdoc. J. 4, 35–50. 10.14304/SURYA.JPR.V4N7.5 27570789 PMC4995773

[B28] Chiappetta D. A. Facorro G. de Celis E. R. Sosnik A. (2011). Synergistic encapsulation of the anti-HIV agent efavirenz within mixed poloxamine/poloxamer polymeric micelles. Nanomedicine 7, 624–637. 10.1016/j.nano.2011.01.017 21371572

[B29] Chirkov S. N. (2002). The antiviral activity of chitosan. Appl. Biochem. Microbiol. 38, 1–8. 10.1023/A:1013206517442 11852567

[B30] Chowdhury M. Y. E. Kim T. H. Uddin M. B. Kim J. H. Hewawaduge C. Y. Ferdowshi Z.. (2017). Mucosal vaccination of conserved sM2, HA2 and cholera toxin subunit A1 (CTA1) fusion protein with poly gamma-glutamate/chitosan nanoparticles (PC NPs) induces protection against divergent influenza subtypes. Vet. Microbiol. 201, 240–251. 10.1016/j.vetmic.2017.01.020 28284616

[B31] Ciejka J. Wolski K. Nowakowska M. Pyrc K. Szczubiałka K. (2017). Biopolymeric nano/microspheres for selective and reversible adsorption of coronaviruses. Mater. Sci. Eng. C. Mater. Biol. Appl. 76, 735–742. 10.1016/j.msec.2017.03.047 28482585 PMC7126271

[B32] Cosgrove S. E. (2006). The relationship between antimicrobial resistance and patient outcomes: mortality, length of hospital stay, and health care costs. Clin. Infect. Dis. 42 Suppl 2, S82–S89. 10.1086/499406 16355321

[B33] Costantino H. R. Illum L. Brandt G. Johnson P. H. Quay S. C. (2007). Intranasal delivery: physicochemical and therapeutic aspects. Int. J. Pharmaceutics 337, 1–24. 10.1016/j.ijpharm.2007.03.025 17475423

[B34] Crisci E. Mussá T. Fraile L. Montoya M. (2013). Influenza virus in pigs. Mol. Immunol. 55, 200–211. 10.1016/j.molimm.2013.02.008 23523121

[B35] Dabaghian M. Latifi A. M. Tebianian M. NajmiNejad H. Ebrahimi S. M. (2018). Nasal vaccination with r4M2e.HSP70c antigen encapsulated into N-trimethyl chitosan (TMC) nanoparticulate systems: Preparation and immunogenicity in a mouse model. Vaccine 36, 2886–2895. 10.1016/j.vaccine.2018.02.072 29627234

[B36] Dai C. Kang H. Yang W. Sun J. Liu C. Cheng G.. (2015). O-2’-hydroxypropyltrimethyl ammonium chloride chitosan nanoparticles for the delivery of live Newcastle disease vaccine. Carbohydr. Polym. 130, 280–289. 10.1016/j.carbpol.2015.05.008 26076628

[B37] das Neves J. Amiji M. M. Bahia M. F. Sarmento B. (2010). Nanotechnology-based systems for the treatment and prevention of HIV/AIDS. Advanced Drug Delivery Rev. 62, 458–477. 10.1016/j.addr.2009.11.017 19914314

[B38] Davidson B. L. McCray P. B. Jr. (2011). Current prospects for RNA interference-based therapies. Nat. Rev. Genet. 12, 329–340. 10.1038/nrg2968 21499294 PMC7097665

[B39] de Britto D. de Moura M. R. Aouada F. A. Mattoso L. H. C. Assis O. B. G. (2012). N, N, N-trimethyl chitosan nanoparticles as a vitamin carrier system. Food Hydrocolloids 27, 487–493. 10.1016/j.foodhyd.2011.09.002

[B40] De Jong M. D. Thanh T. T. Khanh T. H. Hien V. M. Smith G. J. D. Chau N. V.. (2005). Oseltamivir resistance during treatment of influenza A (H5N1) infection. New Engl. J. Med. 353, 2667–2672. 10.1056/NEJMoa054512 16371632

[B41] Dehghan S. Tafaghodi M. Bolourieh T. Mazaheri V. Torabi A. Abnous K.. (2014). Rabbit nasal immunization against influenza by dry-powder form of chitosan nanospheres encapsulated with influenza whole virus and adjuvants. Int. J. Pharm. 475, 1–8. 10.1016/j.ijpharm.2014.08.032 25148732

[B42] Deng X. Cao M. Zhang J. Hu K. Yin Z. Zhou Z.. (2014). Hyaluronic acid-chitosan nanoparticles for co-delivery of MiR-34a and doxorubicin in therapy against triple negative breast cancer. Biomaterials 35, 4333–4344. 10.1016/j.biomaterials.2014.02.006 24565525

[B43] Dhakal S. Renu S. Ghimire S. Shaan Lakshmanappa Y. Hogshead B. T. Feliciano-Ruiz N.. (2018). Mucosal Immunity and Protective Efficacy of Intranasal Inactivated Influenza Vaccine Is Improved by Chitosan Nanoparticle Delivery in Pigs. Front. Immunol. 9, 934. 10.3389/fimmu.2018.00934 29770135 PMC5940749

[B44] Divya K. Jisha M. S. (2018). Chitosan nanoparticles preparation and applications. Environ. Chem. Lett. 16, 101–112. 10.1007/s10311-017-0670-y

[B45] Donalisio M. Leone F. Civra A. Spagnolo R. Ozer O. Lembo D.. (2018). Acyclovir-loaded chitosan nanospheres from nano-emulsion templating for the topical treatment of herpesviruses infections. Pharmaceutics 10, 46. 10.3390/pharmaceutics10020046 PMC6027529 29642603

[B46] D’Affronte L. Platia C.L. (2020). Overview of Infectious Diseases of Concern to Dental Practitioners: Other Viral Infections. In: DePaola L. Grant L. (eds) Infection Control in the Dental Office. (Cham: Springer). 10.1007/978-3-030-30085-2_3

[B47] Dykhuis-Haden C. Painter T. Fangman T. Holtkamp D. (2012). Assessing production parameters and economic impact of swine influenza, PRRS and Mycoplasma hyopneumoniae on finishing pigs in a large production system. Denver: Am. Assoc. Swine Veterinarians, 75–76.

[B48] Einbu A. Vårum K. M. (2008). Characterization of chitin and its hydrolysis to GlcNAc and GlcN. Biomacromolecules 9, 1870–1875. 10.1021/bm8001123 18540645

[B49] El-Shabouri M. H. (2002). Positively charged nanoparticles for improving the oral bioavailability of cyclosporin-A. Int. J. Pharmaceutics 249, 101–108. 10.1016/S0378-5173(02)00461-1 12433438

[B50] Erbacher P. Zou S. Bettinger T. Steffan A.-M. Remy J.-S. (1998). Chitosan-based vector/DNA complexes for gene delivery: biophysical characteristics and transfection ability. Pharm. Res. 15, 1332–1339. 10.1023/A:1011981000671 9755882

[B51] Franca E. F. Lins R. D. Freitas L. C. G. Straatsma T. P. (2008). Characterization of chitin and chitosan molecular structure in aqueous solution. J. Chem. Theory Comput. 4, 2141–2149. 10.1021/ct8002964 26620485

[B52] Funkhouser J. D. Aronson N. N. (2007). Chitinase family GH18: evolutionary insights from the genomic history of a diverse protein family. BMC Evol. Biol. 7, 96. 10.1186/1471-2148-7-96 17594485 PMC1945033

[B53] Gabizon A. Catane R. Uziely B. Kaufman B. Safra T. Cohen R.. (1994). Prolonged circulation time and enhanced accumulation in malignant exudates of doxorubicin encapsulated in polyethylene-glycol coated liposomes. Cancer Res. 54, 987–992. 8313389

[B54] Gabor F. Schwarzbauer A. Wirth M. (2002). Lectin-mediated drug delivery: binding and uptake of BSA-WGA conjugates using the Caco-2 model. Int. J. Pharmaceutics 237, 227–239. 10.1016/S0378-5173(02)00049-2 11955820

[B55] Gagliardi M. (2017). Biomimetic and bioinspired nanoparticles for targeted drug delivery. Ther. Delivery 8, 289–299. 10.4155/tde-2017-0013 28361608

[B56] Gao Y. Liu W. Wang W. Zhang X. Zhao X. (2018). The inhibitory effects and mechanisms of 3, 6-O-sulfated chitosan against human papillomavirus infection. Carbohydr. Polymers 198, 329–338. 10.1016/j.carbpol.2018.06.096 30093007

[B57] Garg U. Chauhan S. Nagaich U. Jain N. (2019). Current advances in chitosan nanoparticles based drug delivery and targeting. Adv. Pharm. Bull. 9, 195. 10.15171/apb.2019.023 31380245 PMC6664124

[B58] Giri N. Tomar P. Karwasara V. S. Pandey R. S. Dixit V. K. (2011). Targeted novel surface-modified nanoparticles for interferon delivery for the treatment of hepatitis B. Acta Biochim. Biophys. Sin. 43, 877–883. 10.1093/abbs/gmr082 21914636

[B59] Glück R. Mischler R. Durrer P. Fürer E. Lang A. B. Herzog C.. (2000). Safety and immunogenicity of intranasally administered inactivated trivalent virosome-formulated influenza vaccine containing Eschevichia coli Heat-Labile Toxin as a Mucosal Adjuvant. J. Infect. Dis. 181, 1129–1132. 10.1086/315337 10720540

[B60] Goldberg M. Langer R. Jia X. (2007). Nanostructured materials for applications in drug delivery and tissue engineering. J. Biomater. Sci. Polym. Ed 18, 241–268. 10.1163/156856207779996931 17471764 PMC3017754

[B61] Gupta P. N. Mahor S. Rawat A. Khatri K. Goyal A. Vyas S. P. (2006). Lectin anchored stabilized biodegradable nanoparticles for oral immunization: 1. Development and *in vitro* evaluation. Int. J. Pharmaceutics 318, 163–173. 10.1016/j.ijpharm.2006.03.017 16621367

[B62] Gupta N. K. Tomar P. Sharma V. Dixit V. K. (2011). Development and characterization of chitosan coated poly-(ε-caprolactone) nanoparticulate system for effective immunization against influenza. Vaccine 29, 9026–9037. 10.1016/j.vaccine.2011.09.033 21939718

[B63] Gupta U. Jain N. K. (2010). Non-polymeric nano-carriers in HIV/AIDS drug delivery and targeting. Advanced Drug Delivery Rev. 62, 478–490. 10.1016/j.addr.2009.11.018 19913579

[B64] Hagenaars N. Mastrobattista E. Verheul R. J. Mooren I. Glansbeek H. L. Heldens J. G. M.. (2009a). Physicochemical and immunological characterization of N, N, N-trimethyl chitosan-coated whole inactivated influenza virus vaccine for intranasal administration. Pharm. Res. 26, 1353–1364. 10.1007/s11095-009-9845-y 19224344

[B65] Hagenaars N. Verheul R. J. Mooren I. de Jong P. H. Mastrobattista E. Glansbeek H. L.. (2009b). Relationship between structure and adjuvanticity of N,N,N-trimethyl chitosan (TMC) structural variants in a nasal influenza vaccine. J. Control Release 140, 126–133. 10.1016/j.jconrel.2009.08.018 19712713

[B66] Hasanovic A. Zehl M. Reznicek G. Valenta C. (2009). Chitosan-tripolyphosphate nanoparticles as a possible skin drug delivery system for aciclovir with enhanced stability. J. Pharm. Pharmacol. 61, 1609–1616. 10.1211/jpp.61.12.0004 19958582

[B67] Hayden F. (2009). Developing new antiviral agents for influenza treatment: what does the future hold? Clin. Infect. Dis. 48 Suppl 1, S3–13. 10.1086/591851 19067613

[B68] He X. Xing R. Liu S. Qin Y. Li K. Yu H.. (2019). The improved antiviral activities of amino-modified chitosan derivatives on Newcastle virus. Drug Chem. Toxicol., 1–6. 10.1080/01480545.2019.1620264 31179762

[B69] Hiremath J. Kang K.-i. Xia M. Elaish M. Binjawadagi B. Ouyang K.. (2016). Entrapment of H1N1 influenza virus derived conserved peptides in PLGA nanoparticles enhances T cell response and vaccine efficacy in pigs. PloS One 11, e0151922. 10.1371/journal.pone.0151922 27093541 PMC4836704

[B70] Hu L. Sun Y. Wu Y. (2013). Advances in chitosan-based drug delivery vehicles. Nanoscale 5, 3103–3111. 10.1039/c3nr00338h 23515527

[B71] Huang H. N. Li T. L. Chan Y. L. Chen C. L. Wu C. J. (2009). Transdermal immunization with low-pressure-gene-gun mediated chitosan-based DNA vaccines against Japanese encephalitis virus. Biomaterials 30, 6017–6025. 10.1016/j.biomaterials.2009.07.029 19656560

[B72] Hunsawong T. Sunintaboon P. Warit S. Thaisomboonsuk B. Jarman R. G. Yoon I. K.. (2015). Immunogenic Properties of a BCG Adjuvanted Chitosan Nanoparticle-Based Dengue Vaccine in Human Dendritic Cells. PloS Negl. Trop. Dis. 9, e0003958. 10.1371/journal.pntd.0003958 26394138 PMC4578877

[B73] Ilium L. (1998). Chitosan and its use as a pharmaceutical excipient. Pharm. Res. 15, 1326–1331. 10.1023/A:1011929016601 9755881

[B74] Illum L. (2003). Nasal drug delivery—possibilities, problems and solutions. J. Controlled Release 87, 187–198. 10.1016/S0168-3659(02)00363-2 12618035

[B75] Ingle A. Gade A. Pierrat S. Sonnichsen C. Rai M. (2008). Mycosynthesis of silver nanoparticles using the fungus Fusarium acuminatum and its activity against some human pathogenic bacteria. Curr. Nanosci. 4, 141–144. 10.2174/157341308784340804

[B76] Iranpur Mobarakeh V. Modarressi M. H. Rahimi P. Bolhassani A. Arefian E. Atyabi F.. (2019). Optimization of chitosan nanoparticles as an anti-HIV siRNA delivery vehicle. Int. J. Biol. Macromol. 129, 305–315. 10.1016/j.ijbiomac.2019.02.036 30738164

[B77] Ito T. Couceiro J.N. S. S. Kelm S. Baum L. G. Krauss S. Castrucci M. R.. (1998). Molecular basis for the generation in pigs of influenza A viruses with pandemic potential. J. Virol. 72, 7367–7373. 10.1128/JVI.72.9.7367-7373.1998 9696833 PMC109961

[B78] Jamali A. Mottaghitalab F. Abdoli A. Dinarvand M. Esmailie A. Kheiri M. T.. (2018). Inhibiting influenza virus replication and inducing protection against lethal influenza virus challenge through chitosan nanoparticles loaded by siRNA. Drug Delivery Transl. Res. 8, 12–20. 10.1007/s13346-017-0426-z 29063498

[B79] Jaworska M. M. Antos D. Górak A. (2020). Review on the application of chitin and chitosan in chromatography. Reactive Funct. Polymers, 104606. 10.1016/j.reactfunctpolym.2020.104606

[B80] Je J.-Y. Kim S.-K. (2006). Reactive oxygen species scavenging activity of aminoderivatized chitosan with different degree of deacetylation. Bioorg. medicinal Chem. 14, 5989–5994. 10.1016/j.bmc.2006.05.016 16725329

[B81] Jiang L. Qian F. He X. Wang F. Ren D. He Y.. (2007). Novel chitosan derivative nanoparticles enhance the immunogenicity of a DNA vaccine encoding hepatitis B virus core antigen in mice. J. Gene Med. 9, 253–264. 10.1002/jgm.1017 17397104

[B82] Jin Z. Li D. Dai C. Cheng G. Wang X. Zhao K. (2017). Response of live Newcastle disease virus encapsulated in N-2-hydroxypropyl dimethylethyl ammonium chloride chitosan nanoparticles. Carbohydr. Polym. 171, 267–280. 10.1016/j.carbpol.2017.05.022 28578963

[B83] Jonassen H. Kjøniksen A.-L. Hiorth M. (2012). Stability of chitosan nanoparticles cross-linked with tripolyphosphate. Biomacromolecules 13, 3747–3756. 10.1021/bm301207a 23046433

[B84] Kabanov A. V. Bronich T. K. Kabanov V. A. Yu K. Eisenberg A. (1996). Soluble stoichiometric complexes from poly (N-ethyl-4-vinylpyridinium) cations and poly (ethylene oxide)-block-polymethacrylate anions. Macromolecules 29, 6797–6802. 10.1021/ma960120k

[B85] Kang M. L. Cho C. S. Yoo H. S. (2009). Application of chitosan microspheres for nasal delivery of vaccines. Biotechnol. Adv. 27, 857–865. 10.1016/j.biotechadv.2009.06.007 19583998

[B86] Kato Y. Onishi H. Machida Y. (2001). Biological characteristics of lactosaminated N-succinyl-chitosan as a liver-specific drug carrier in mice. J. Controlled Release 70, 295–307. 10.1016/S0168-3659(00)00356-4 11182200

[B87] Kavaliauskis A. Arnemo M. Speth M. Lagos L. Rishovd A. L. Estepa A.. (2016). Protective effect of a recombinant VHSV-G vaccine using poly(I:C) loaded nanoparticles as an adjuvant in zebrafish (Danio rerio) infection model. Dev. Comp. Immunol. 61, 248–257. 10.1016/j.dci.2016.04.010 27084059

[B88] Khatri K. Goyal A. K. Gupta P. N. Mishra N. Mehta A. Vyas S. P. (2008a). Surface modified liposomes for nasal delivery of DNA vaccine. Vaccine 26, 2225–2233. 10.1016/j.vaccine.2008.02.058 18396362

[B89] Khatri K. Goyal A. K. Gupta P. N. Mishra N. Vyas S. P. (2008b). Plasmid DNA loaded chitosan nanoparticles for nasal mucosal immunization against hepatitis B. Int. J. Pharm. 354, 235–241. 10.1016/j.ijpharm.2007.11.027 18182259

[B90] Khimmakthong U. Kongmee P. Deachamag P. Leggat U. Chotigeat W. (2013). Activation of an immune response in Litopenaeus vannamei by oral immunization with phagocytosis activating protein (PAP) DNA. Fish Shellfish Immunol. 34, 929–938. 10.1016/j.fsi.2013.01.004 23353001

[B91] Kim A. E. Dintaman J. M. Waddell D. S. Silverman J. A. (1998). Saquinavir, an HIV protease inhibitor, is transported by P-glycoprotein. J. Pharmacol. Exp. Ther. 286, 1439–1445. 9732409

[B92] Kim R. B. Fromm M. F. Wandel C. Leake B. Wood A. J. Roden D. M.. (1998). The drug transporter P-glycoprotein limits oral absorption and brain entry of HIV-1 protease inhibitors. J. Clin. Invest. 101, 289–294. 10.1172/JCI1269 9435299 PMC508566

[B93] Kim S.-K. (2013). Marine biomaterials: characterization, isolation and applications (CRC press).

[B94] Kim S.-H. Jang Y.-S. (2017). The development of mucosal vaccines for both mucosal and systemic immune induction and the roles played by adjuvants. Clin. Exp. Vaccine Res. 6, 15–21. 10.7774/cevr.2017.6.1.15 28168169 PMC5292352

[B95] Kim B. G. Kang I. J. (2008). Evaluation of the effects of biodegradable nanoparticles on a vaccine delivery system using AFM, SEM, and TEM. Ultramicroscopy 108, 1168–1173. 10.1016/j.ultramic.2008.04.038 18554804

[B96] Klaykruayat B. Siralertmukul K. Srikulkit K. (2010). Chemical modification of chitosan with cationic hyperbranched dendritic polyamidoamine and its antimicrobial activity on cotton fabric. Carbohydr. Polymers 80, 197–207. 10.1016/j.carbpol.2009.11.013

[B97] Kmiec M. Pighinelli L. Tedesco M. F. Silva M. M. Reis V. (2017). Chitosan-properties and applications in dentistry. Adv. Tissue Eng. Regener. Med. Open Access 2, 00035.

[B98] Kole R. Krainer A. R. Altman S. (2012). RNA therapeutics: beyond RNA interference and antisense oligonucleotides. Nat. Rev. Drug Discovery 11, 125–140. 10.1038/nrd3625 22262036 PMC4743652

[B99] Kole S. Qadiri S. S. N. Shin S. M. Kim W. S. Lee J. Jung S. J. (2019). Nanoencapsulation of inactivated-viral vaccine using chitosan nanoparticles: Evaluation of its protective efficacy and immune modulatory effects in olive flounder (Paralichthys olivaceus) against viral haemorrhagic septicaemia virus (VHSV) infection. Fish Shellfish Immunol. 91, 136–147. 10.1016/j.fsi.2019.05.017 31096061

[B100] Kulikov S. N. Chirkov S. N. Il’ina A. V. Lopatin S. A. Varlamov V. P. (2006). Effect of the molecular weight of chitosan on its antiviral activity in plants. Appl. Biochem. Microbiol. 42, 200–203. 10.1134/S0003683806020165 16761579

[B101] Kumar M. N. Muzzarelli R. A. Muzzarelli C. Sashiwa H. Domb A. J. (2004). Chitosan chemistry and pharmaceutical perspectives. Chem. Rev. 104, 6017–6084. 10.1021/cr030441b 15584695

[B102] Kumar A. Ma H. Zhang X. Huang K. Jin S. Liu J.. (2012). Gold nanoparticles functionalized with therapeutic and targeted peptides for cancer treatment. Biomaterials 33, 1180–1189. 10.1016/j.biomaterials.2011.10.058 22056754

[B103] Kwon S. Y. Lee C. H. (2011). Epidemiology and prevention of hepatitis B virus infection. Korean J. Hepatol. 17, 87. 10.3350/kjhep.2011.17.2.87 21757978 PMC3304633

[B104] Kyd J. M. Ruth Foxwell A. Cripps A. W. (2001). Mucosal immunity in the lung and upper airway. Vaccine 19, 2527–2533. 10.1016/S0264-410X(00)00484-9 11257388

[B105] Langer R. (1998). Drug delivery and targeting. Nature 392, 5–10. 9579855

[B106] Lara H. H. Ayala-Nuñez N. V. Ixtepan-Turrent L. Rodriguez-Padilla C. (2010). Mode of antiviral action of silver nanoparticles against HIV-1. J. Nanobiotechnol. 8, 1. 10.1186/1477-3155-8-1 PMC2818642 20145735

[B107] Lavelle E. C. Grant G. Pfuller U. O’Hagan D. T. (2004). Immunological implications of the use of plant lectins for drug and vaccine targeting to the gastrointestinal tract. J. Drug Targeting 12, 89–95. 10.1080/10611860410001693733 15203902

[B108] Le Cleach L. Trinquart L. Do G. Maruani A. Lebrun-Vignes B. Ravaud P.. (2014). Oral antiviral therapy for prevention of genital herpes outbreaks in immunocompetent and nonpregnant patients. Cochrane Database System. Rev. 3 (8), CD009036. 10.1002/14651858.CD009036.pub2 PMC11022119 25086573

[B109] Lee J. Kim Y. M. Kim J. H. Cho C. W. Jeon J. W. Park J. K.. (2018). Nasal delivery of chitosan/alginate nanoparticle encapsulated bee (Apis mellifera) venom promotes antibody production and viral clearance during porcine reproductive and respiratory syndrome virus infection by modulating T cell related responses. Vet. Immunol. Immunopathol. 200, 40–51. 10.1016/j.vetimm.2018.04.006 29776611

[B110] Lembo D. Donalisio M. Rusnati M. Bugatti A. Cornaglia M. Cappello P.. (2008). Sulfated K5 Escherichia coli polysaccharide derivatives as wide-range inhibitors of genital types of human papillomavirus. Antimicrob. Agents Chemother. 52, 1374–1381. 10.1128/AAC.01467-07 18250186 PMC2292566

[B111] Li Q. Du Y.-Z. Yuan H. Zhang X.-G. Miao J. Cui F.-D.. (2010). Synthesis of lamivudine stearate and antiviral activity of stearic acid-g-chitosan oligosaccharide polymeric micelles delivery system. Eur. J. Pharm. Sci. 41, 498–507. 10.1016/j.ejps.2010.08.004 20728535

[B112] Li X. Wu P. Gao G. F. Cheng S. (2011). Carbohydrate-functionalized chitosan fiber for influenza virus capture. Biomacromolecules 12, 3962–3969. 10.1021/bm200970x 21978096

[B113] Li X. Chan W. K. (1999). Transport, metabolism and elimination mechanisms of anti-HIV agents. Advanced Drug Delivery Rev. 39, 81–103. 10.1016/S0169-409X(99)00021-6 10837769

[B114] Lin A. Liu Y. Huang Y. Sun J. Wu Z. Zhang X.. (2008). Glycyrrhizin surface-modified chitosan nanoparticles for hepatocyte-targeted delivery. Int. J. Pharmaceutics 359, 247–253. 10.1016/j.ijpharm.2008.03.039 18457928

[B115] Little S. J. Holte S. Routy J.-P. Daar E. S. Markowitz M. Collier A. C.. (2002). Antiretroviral-drug resistance among patients recently infected with HIV. New Engl. J. Med. 347, 385–394. 10.1056/NEJMoa013552 12167680

[B116] Liu Q. Zheng X. Zhang C. Shao X. Zhang X. Zhang Q.. (2015). Conjugating influenza a (H1N1) antigen to n-trimethylaminoethylmethacrylate chitosan nanoparticles improves the immunogenicity of the antigen after nasal administration. J. Med. Virol. 87, 1807–1815. 10.1002/jmv.24253 25959372

[B117] Lockhat H. A. Silva J. R. A. Alves C. N. Govender T. Lameira J. Maguire G. E. M.. (2016). Binding free energy calculations of nine FDA-approved protease inhibitors against HIV-1 subtype C I36T↑ T containing 100 amino acids per monomer. Chem. Biol. Drug design 87, 487–498. 10.1111/cbdd.12690 26613568

[B118] Lopes P. D. Okino C. H. Fernando F. S. Pavani C. Casagrande V. M. Lopez R. F. V.. (2018). Inactivated infectious bronchitis virus vaccine encapsulated in chitosan nanoparticles induces mucosal immune responses and effective protection against challenge. Vaccine 36, 2630–2636. 10.1016/j.vaccine.2018.03.065 29653848

[B119] Louw A. M. Kolar M. K. Novikova L. N. Kingham P. J. Wiberg M. Kjems J.. (2016). Chitosan polyplex mediated delivery of miRNA-124 reduces activation of microglial cells *in vitro* and in rat models of spinal cord injury. Nanomedicine 12, 643–653. 10.1016/j.nano.2015.10.011 26582736

[B120] Lugade A. A. Bharali D. J. Pradhan V. Elkin G. Mousa S. A. Thanavala Y. (2013). Single low-dose un-adjuvanted HBsAg nanoparticle vaccine elicits robust, durable immunity. Nanomedicine 9, 923–934. 10.1016/j.nano.2013.03.008 23542018

[B121] Luo Y. Wang Q. (2014). Recent development of chitosan-based polyelectrolyte complexes with natural polysaccharides for drug delivery. Int. J. Biol. Macromol. 64, 353–367. 10.1016/j.ijbiomac.2013.12.017 24360899

[B122] Maitra A. Ghosh P. K. De T. K. Sahoo S. K. (1999). “Process for the preparation of highly monodispersed polymeric hydrophilic nanoparticles,” in Google Patents.

[B123] Mallipeddi R. Rohan L. C. (2010). Progress in antiretroviral drug delivery using nanotechnology. Int. J. Nanomed. 5, 533–547. PMC2950411 20957115

[B124] Maseko S. B. Natarajan S. Sharma V. Bhattacharyya N. Govender T. Sayed Y. (2016). Purification and characterization of naturally occurring HIV-1 (South African subtype C) protease mutants from inclusion bodies. Protein Expression Purification 122, 90–96. 10.1016/j.pep.2016.02.013 26917227

[B125] McKiernan P. J. Greene C. M. (2015). MicroRNA Dysregulation in Cystic Fibrosis. Mediators Inflammation 2015, 529642. 10.1155/2015/529642 PMC4491587 26185362

[B126] McNeil S. E. (2011). Unique benefits of nanotechnology to drug delivery and diagnostics. Methods Mol. Biol. 697, 3–8. 10.1007/978-1-60327-198-1_1 21116949

[B127] Medepalli K. K. (2008). Advanced nanomaterials for biomedical applications (University of Louisville).

[B128] Mehendale R. Joshi M. Patravale V. B. (2013). Nanomedicines for treatment of viral diseases. Crit. Rev. Ther. Drug Carrier Syst. 30, 1–49. 10.1615/CritRevTherDrugCarrierSyst.2013005469 23510109

[B129] Meng X. Xing R. Liu S. Yu H. Li K. Qin Y.. (2012). Molecular weight and pH effects of aminoethyl modified chitosan on antibacterial activity *in vitro* . Int. J. Biol. Macromol. 50, 918–924. 10.1016/j.ijbiomac.2012.01.018 22342739

[B130] Meng J. Zhang T. Agrahari V. Ezoulin M. J. Youan B. B. (2014). Comparative biophysical properties of tenofovir-loaded, thiolated and nonthiolated chitosan nanoparticles intended for HIV prevention. Nanomed. (Lond) 9, 1595–1612. 10.2217/nnm.13.136 PMC4278848 24405490

[B131] Mishra N. Khatri K. Gupta M. Vyas S. P. (2014). Development and characterization of LTA-appended chitosan nanoparticles for mucosal immunization against hepatitis B. Artif. Cells Nanomed. Biotechnol. 42, 245–255. 10.3109/21691401.2013.809726 23815286

[B132] Mohamed S. H. Arafa A. S. Mady W. H. Fahmy H. A. Omer L. M. Morsi R. E. (2018). Preparation and immunological evaluation of inactivated avian influenza virus vaccine encapsulated in chitosan nanoparticles. Biologicals 51, 46–53. 10.1016/j.biologicals.2017.10.004 29126666

[B133] Mohammed M. A. Syeda J. Wasan K. M. Wasan E. K. (2017). An overview of chitosan nanoparticles and its application in non-parenteral drug delivery. Pharmaceutics 9, 53. 10.3390/pharmaceutics9040053 PMC5750659 29156634

[B134] Moon H. J. Lee J. S. Talactac M. R. Chowdhury M. Y. Kim J. H. Park M. E.. (2012). Mucosal immunization with recombinant influenza hemagglutinin protein and poly gamma-glutamate/chitosan nanoparticles induces protection against highly pathogenic influenza A virus. Vet. Microbiol. 160, 277–289. 10.1016/j.vetmic.2012.05.035 22763171

[B135] Moreno P. M. Pêgo A. P. (2014). Therapeutic antisense oligonucleotides against cancer: hurdling to the clinic. Front. Chem. 2, 87. 10.3389/fchem.2014.00087 25353019 PMC4196572

[B136] Muthu M. S. Singh S. (2009). Targeted nanomedicines: effective treatment modalities for cancer, AIDS and brain disorders. Nanomed. (Lond) 4, 105–118. 10.2217/17435889.4.1.105 19093899

[B137] Muzzarelli R. A. A. Jeuniaux C. Gooday G. W. (1986). Chitin in nature and technology (Springer).

[B138] Muzzarelli R. A. A. Mattioli-Belmonte M. Muzzarelli B. (1998). Advances in Chitin. SciLyon: Andru 2, 219–224.

[B139] Nagpal K. Singh S. K. Mishra D. N. (2010). Chitosan nanoparticles: a promising system in novel drug delivery. Chem. Pharm. Bull. 58, 1423–1430. 10.1248/cpb.58.1423 21048331

[B140] Nahand J. S. Jamshidi S. Hamblin M. R. Mahjoubin-Tehran M. Vosough M. Jamali M.. (2020a). Circular RNAs: New Epigenetic Signatures in Viral Infections. Front. Microbiol. 11, 1853. 10.3389/fmicb.2020.01853 32849445 PMC7412987

[B141] Nahand J. S. Mahjoubin-Tehran M. Moghoofei M. Pourhanifeh M. H. Mirzaei H. R. Asemi Z.. (2020b). Exosomal miRNAs: novel players in viral infection. Epigenomics 12, 353–370. 10.2217/epi-2019-0192 32093516 PMC7713899

[B142] Nalwa H. S. (1999). Handbook of nanostructured materials and nanotechnology, five-volume set (Academic Press).

[B143] Nanda R. K. Hajam I. A. Edao B. M. Ramya K. Rajangam M. Chandra Sekar S.. (2014). Immunological evaluation of mannosylated chitosan nanoparticles based foot and mouth disease virus DNA vaccine, pVAC FMDV VP1-OmpA in guinea pigs. Biologicals 42, 153–159. 10.1016/j.biologicals.2014.01.002 24656961

[B144] Nasrollahzadeh M. Shafiei N. Nezafat Z. Bidgoli N. S. S. Soleimani F. (2020). Recent progresses in the application of cellulose, starch, alginate, gum, pectin, chitin and chitosan based (nano) catalysts in sustainable and selective oxidation reactions: A review. Carbohydr. Polymers, 116353. 10.1016/j.carbpol.2020.116353 32507224

[B145] Neutra M. R. Kozlowski P. A. (2006). Mucosal vaccines: the promise and the challenge. Nat. Rev. Immunol. 6, 148–158. 10.1038/nri1777 16491139

[B146] Ng J. C. Y. Cheung W. H. McKay G. (2002). Equilibrium studies of the sorption of Cu (II) ions onto chitosan. J. Colloid Interface Sci. 255, 64–74. 10.1006/jcis.2002.8664 12702369

[B147] Ngo D.-H. Vo T.-S. Ngo D.-N. Kang K.-H. Je J.-Y. Pham H. N.-D.. (2015). Biological effects of chitosan and its derivatives. Food Hydrocolloids 51, 200–216. 10.1016/j.foodhyd.2015.05.023

[B148] Niemeyer C. M. (2006). Nanobiotechnology. Rev. Cell Biol. Mol. Med.

[B149] Nishiyama N. Kato Y. Sugiyama Y. Kataoka K. (2001). Cisplatin-loaded polymer-metal complex micelle with time-modulated decaying property as a novel drug delivery system. Pharm. Res. 18, 1035–1041. 10.1023/A:1010908916184 11496942

[B150] Niwa T. Takeuchi H. Hino T. Kunou N. Kawashima Y. (1993). Preparations of biodegradable nanospheres of water-soluble and insoluble drugs with D, L-lactide/glycolide copolymer by a novel spontaneous emulsification solvent diffusion method and the drug release behavior. J. Controlled Release 25, 89–98. 10.1016/0168-3659(93)90097-O

[B151] Nugent J. (1998). The design and delivery of non-parenteral vaccines. 9867311

[B152] Pan L. Zhang Z. Lv J. Zhou P. Hu W. Fang Y.. (2014). Induction of mucosal immune responses and protection of cattle against direct-contact challenge by intranasal delivery with foot-and-mouth disease virus antigen mediated by nanoparticles. Int. J. Nanomed. 9, 5603–5618. 10.2147/IJN.S72318 PMC4260661 25506214

[B153] Panácek A. Kolár M. Vecerová R. Prucek R. Soukupová J. Krystof V.. (2009). Antifungal activity of silver nanoparticles against Candida spp. Biomaterials 30, 6333–6340. 10.1016/j.biomaterials.2009.07.065 19698988

[B154] Parboosing R. Maguire G. E. Govender P. Kruger H. G. (2012). Nanotechnology and the treatment of HIV infection. Viruses 4, 488–520. 10.3390/v4040488 22590683 PMC3347320

[B155] Pathinayake P. S. Gayan Chathuranga W. A. Lee H. C. Chowdhury M. Y. E. Sung M. H. Lee J. S.. (2018). Inactivated enterovirus 71 with poly-γ-glutamic acid/Chitosan nano particles (PC NPs) induces high cellular and humoral immune responses in BALB/c mice. Arch. Virol. 163, 2073–2083. 10.1007/s00705-018-3837-3 29619599

[B156] Pawar R. Jadhav W. Bhusare S. Borade R. Farber S. Itzkowitz D.. (2008). “Polysaccharides as carriers of bioactive agents for medical applications,” in Natural-based polymers for biomedical applications (Elsevier).

[B157] Peter M. G. (1995). Applications and environmental aspects of chitin and chitosan. J. Macromol. Sci. Part A: Pure Appl. Chem. 32, 629–640.

[B158] Petros R. A. DeSimone J. M. (2010). Strategies in the design of nanoparticles for therapeutic applications. Nat. Rev. Drug Discovery 9, 615–627. 10.1038/nrd2591 20616808

[B159] Pillai C. K. S. Paul W. Sharma C. P. (2009). Chitin and chitosan polymers: Chemistry, solubility and fiber formation. Prog. Polymer Sci. 34, 641–678. 10.1016/j.progpolymsci.2009.04.001

[B160] Poo H. Park C. Kwak M.-S. Choi D.-Y. Hong S.-P. Lee I.-H.. (2010). New Biological Functions and Applications of High-Molecular-Mass Poly-γ-glutamic Acid. Chem. Biodiversity 7, 1555–1562. 10.1002/cbdv.200900283 20564573

[B161] Pospieszny H. Chirkov S. Atabekov J. (1991). Induction of antiviral resistance in plants by chitosan. Plant Sci. 79, 63–68. 10.1016/0168-9452(91)90070-O

[B162] Prabhu P. Patravale V. Joshi M. (2012). Nanocarriers for effective topical delivery of anti-infectives. Curr. Nanosci. 8, 491–503. 10.2174/157341312801784221

[B163] Qasim M. Lim D. J. Park H. Na D. (2014). Nanotechnology for diagnosis and treatment of infectious diseases. J. Nanosci. Nanotechnol. 14, 7374–7387. 10.1166/jnn.2014.9578 25942798

[B164] Qi M. Zhang X.-E. Sun X. Zhang X. Yao Y. Liu S.. (2018). Intranasal nanovaccine confers homo-and hetero-subtypic influenza protection. Small 14, 1703207. 10.1002/smll.201703207 29430819

[B165] Radhakumary C. Antonty M. Sreenivasan K. (2011). Drug loaded thermoresponsive and cytocompatible chitosan based hydrogel as a potential wound dressing. Carbohydr. Polymers 83, 705–713. 10.1016/j.carbpol.2010.08.042

[B166] Ragelle H. Vandermeulen G. Préat V. (2013). Chitosan-based siRNA delivery systems. J. Control Release 172, 207–218. 10.1016/j.jconrel.2013.08.005 23965281

[B167] Ramana L. N. Sharma S. Sethuraman S. Ranga U. Krishnan U. M. (2014). Evaluation of chitosan nanoformulations as potent anti-HIV therapeutic systems. Biochim. Biophys. Acta 1840, 476–484. 10.1016/j.bbagen.2013.10.002 24121104

[B168] Rani D. Saxena R. Nayak B. Srivastava S. (2018). Cloning and expression of truncated ORF2 as a vaccine candidate against hepatitis E virus 8 (10), 414. 10.1007/s13205-018-1437-2 PMC6139098 30237961

[B169] Rashki S. Asgarpour K. Tarrahimofrad H. Hashemipour M. Ebrahimi M. S. Fathizadeh H.. (2020). Chitosan-Based Nanoparticles against bacterial infections. Carbohydr. Polymers, 117108. 10.1016/j.carbpol.2020.117108 33142645

[B170] Rashki S. Asgarpour K. Tarrahimofrad H. Hashemipour M. Ebrahimi M. S. Fathizadeh H.. (2021). Chitosan-based nanoparticles against bacterial infections. Carbohydr. Polym. 251, 117108. 10.1016/j.carbpol.2020.117108 33142645

[B171] Roizman B. (2006). Herpes simplex virus. Fields Virol., 2501–2601.

[B172] ROUGET C. (1859). Des substances anlylacees duns Ie tissu des aninltUIX specialenlcnt [esurticules (Chitine). ()nlptes Rendus 48t, 792–795.

[B173] Sadati S. F. Jamali A. Abdoli A. Abedi-Valugerdi M. Gholami S. Alipour S.. (2018). Simultaneous formulation of influenza vaccine and chitosan nanoparticles within CpG oligodesoxi nucleotides leads to dose-sparing and protects against lethal challenge in the mouse model. Pathog. Dis. 76. 10.1093/femspd/fty070 30184220

[B174] Sadri Nahand J. Bokharaei-Salim F. Karimzadeh M. Moghoofei M. Karampoor S. Mirzaei H. R.. (2020). MicroRNAs and exosomes: key players in HIV pathogenesis. HIV Med. 21, 246–278. 10.1111/hiv.12822 31756034 PMC7069804

[B175] Saikia C. Gogoi P. Maji T. K. (2015). Chitosan: A promising biopolymer in drug delivery applications. J. Mol. Genet. Med. S 4. 10.4172/1747-0862.S4-006

[B176] Santos-Carballal B. Fernández Fernández E. Goycoolea F. M. (2018). Chitosan in Non-Viral Gene Delivery: Role of Structure, Characterization Methods, and Insights in Cancer and Rare Diseases Therapies. Polymers (Basel) 10. 10.3390/polym10040444 PMC6415274 30966479

[B177] Santos-Martinez M. J. Rahme K. Corbalan J. J. Faulkner C. Holmes J. D. Tajber L.. (2014). Pegylation increases platelet biocompatibility of gold nanoparticles. J. BioMed. Nanotechnol. 10, 1004–1015. 10.1166/jbn.2014.1813 24749395

[B178] Sanvicens N. Marco M. P. (2008). Multifunctional nanoparticles–properties and prospects for their use in human medicine. Trends Biotechnol. 26, 425–433. 10.1016/j.tibtech.2008.04.005 18514941

[B179] Sawaengsak C. Mori Y. Yamanishi K. Mitrevej A. Sinchaipanid N. (2014). Chitosan nanoparticle encapsulated hemagglutinin-split influenza virus mucosal vaccine. AAPS PharmSciTech 15, 317–325. 10.1208/s12249-013-0058-7 24343789 PMC3969489

[B180] Schapiro J. M. Winters M. A. Stewart F. Efron B. Norris J. Kozal M. J.. (1996). The effect of high-dose saquinavir on viral load and CD4+ T-cell counts in HIV-infected patients. Ann. Internal Med. 124, 1039–1050. 10.7326/0003-4819-124-12-199606150-00003 8633817

[B181] Scheller F. W. Bier F. F. Pfeiffer D. (1995). Biosensoren: grundlagen und anwendungen. tm-Technisches Messen 62, 213–219. 10.1524/teme.1995.62.jg.213

[B182] Schütz C. A. Juillerat-Jeanneret L. Mueller H. Lynch I. Riediker M. (2013). Therapeutic nanoparticles in clinics and under clinical evaluation. Nanomed. (Lond) 8, 449–467. 10.2217/nnm.13.8 23477336

[B183] Serrano-Sevilla I. Artiga Á. Mitchell S. G. De Matteis L. de la Fuente J. M. (2019). Natural Polysaccharides for siRNA Delivery: Nanocarriers Based on Chitosan, Hyaluronic Acid, and Their Derivatives. Molecules 24. 10.3390/molecules24142570 PMC6680562 31311176

[B184] Shafer R. W. Rhee S.-Y. Pillay D. Miller V. Sandstrom P. Schapiro J. M.. (2007). HIV-1 protease and reverse transcriptase mutations for drug resistance surveillance. AIDS (London England) 21, 215. 10.1097/QAD.0b013e328011e691 PMC2573394 17197813

[B185] Shafti-Keramat S. Handisurya A. Kriehuber E. Meneguzzi G. Slupetzky K. Kirnbauer R. (2003). Different heparan sulfate proteoglycans serve as cellular receptors for human papillomaviruses. J. Virol. 77, 13125–13135. 10.1128/JVI.77.24.13125-13135.2003 14645569 PMC296080

[B186] Sharma V. K. Sharma R. K. Singh S. K. (2014). Antisense oligonucleotides: modifications and clinical trials. MedChemComm 5, 1454–1471. 10.1039/C4MD00184B

[B187] Shibata Y. Foster L.A. Metzger W.J. Myrvik Q. N. (1997). Alveolar macrophage priming by intravenous administration of chitin particles, polymers of N-acetyl-D-glucosamine, in mice. Infect. Immun. 65, 1734–1741. 10.1128/IAI.65.5.1734-1741.1997 9125555 PMC175208

[B188] Singh P. Prabakaran D. Jain S. Mishra V. Jaganathan K. S. Vyas S. P. (2004). Cholera toxin B subunit conjugated bile salt stabilized vesicles (bilosomes) for oral immunization. Int. J. Pharmaceutics 278, 379–390. 10.1016/j.ijpharm.2004.03.014 15196642

[B189] Singh B. Choi Y. J. Park I. K. Akaike T. Cho C. S. (2014). Chemical modification of chitosan with pH-sensitive molecules and specific ligands for efficient DNA transfection and siRNA silencing. J. Nanosci. Nanotechnol. 14, 564–576. 10.1166/jnn.2014.9079 24730283

[B190] Singh R. P. Gangadharappa H. V. Mruthunjaya K. (2018). Phytosome complexed with chitosan for gingerol delivery in the treatment of respiratory infection: In vitro and *in vivo* evaluation. Eur. J. Pharm. Sci. 122, 214–229. 10.1016/j.ejps.2018.06.028 29966737

[B191] Slütter B. Bal S. M. Que I. Kaijzel E. Loüwik C. Bouwstra J.. (2010). Antigen– adjuvant nanoconjugates for nasal vaccination: an improvement over the use of nanoparticles? Mol. Pharmaceutics 7, 2207–2215. 10.1021/mp100210g 21043518

[B192] Spruance S. L. Kriesel J. D. (2002). Treatment of herpes simplex labialis. Herpes: J. IHMF 9, 64–69. 12470603

[B193] Su X. Zivanovic S. D’SOUZA DORIS H (2009). Effect of chitosan on the infectivity of murine norovirus, feline calicivirus, and bacteriophage MS2. J. Food Prot. 72, 2623–2628. 10.4315/0362-028X-72.12.2623 20003751

[B194] Subbiah R. Ramalingam P. Ramasundaram S. Kim D. Y. Park K. Ramasamy M. K.. (2012). N,N,N-Trimethyl chitosan nanoparticles for controlled intranasal delivery of HBV surface antigen. Carbohydr. Polym. 89, 1289–1297. 10.1016/j.carbpol.2012.04.056 24750944

[B195] Sun R. W. Chen R. Chung N. P. Ho C. M. Lin C. L. Che C. M. (2005). Silver nanoparticles fabricated in Hepes buffer exhibit cytoprotective activities toward HIV-1 infected cells. Chem. Commun. (Camb.), 5059–5061. 10.1039/b510984a 16220170

[B196] Tahamtan A. Barati M. Tabarraei A. Mohebbi S. R. Shirian S. Gorji A.. (2018). Antitumor Immunity Induced by Genetic Immunization with Chitosan Nanoparticle Formulated Adjuvanted for HPV-16 E7 DNA Vaccine. Iran J. Immunol. 15, 269–280. 30593741 10.22034/IJI.2018.39396

[B197] Taju G. Kumar D. V. Majeed S. A. Vimal S. Tamizhvanan S. Kumar S. S.. (2018). Delivery of viral recombinant VP28 protein using chitosan tripolyphosphate nanoparticles to protect the whiteleg shrimp, Litopenaeus vannamei from white spot syndrome virus infection. Int. J. Biol. Macromol. 107, 1131–1141. 10.1016/j.ijbiomac.2017.09.094 28951305

[B198] Tamura S.-i. Kurata T. (2004). Defense mechanisms against influenza virus infection in the respiratory tract mucosa. Jpn. J. Infect. Dis. 57, 236–247. 15623947

[B199] Tang R. Zhai Y. Dong L. Malla T. Hu K. (2018). Immunization with dendritic cell-based DNA vaccine pRSC-NLDC145.gD-IL21 protects mice against herpes simplex virus keratitis. Immunotherapy 10, 189–200. 10.2217/imt-2017-0060 29370719

[B200] Tao F. Cheng Y. Shi X. Zheng H. Du Y. Deng H. (2020). Applications of chitin and chitosan nanofibers in bone regenerative engineering. Carbohydr. Polymers 230, 115658. 10.1016/j.carbpol.2019.115658 31887899

[B201] Taranejoo S. Janmaleki M. Rafienia M. Kamali M. Mansouri M. (2011). Chitosan microparticles loaded with exotoxin A subunit antigen for intranasal vaccination against Pseudomonas aeruginosa: An *in vitro* study. Carbohydr. Polymers 83, 1854–1861. 10.1016/j.carbpol.2010.10.051

[B202] Theerawanitchpan G. Saengkrit N. Sajomsang W. Gonil P. Ruktanonchai U. Saesoo S.. (2012). Chitosan and its quaternized derivative as effective long dsRNA carriers targeting shrimp virus in Spodoptera frugiperda 9 cells. J. Biotechnol. 160, 97–104. 10.1016/j.jbiotec.2012.04.011 22575788

[B203] Thomas C. Rawat A. Hope-Weeks L. Ahsan F. (2011). Aerosolized PLA and PLGA nanoparticles enhance humoral, mucosal and cytokine responses to hepatitis B vaccine. Mol. Pharmaceutics 8, 405–415. 10.1021/mp100255c 21189035

[B204] Thummel K. E. Kunze K. L. Shen D. D. (1997). Enzyme-catalyzed processes of first-pass hepatic and intestinal drug extraction. Advanced Drug Delivery Rev. 27, 99–127. 10.1016/S0169-409X(97)00039-2 10837554

[B205] Timur S. S. Şahin A. Aytekin E. Öztürk N. Polat K. H. Tezel N.. (2018). Design and *in vitro* evaluation of tenofovir-loaded vaginal gels for the prevention of HIV infections. Pharm. Dev. Technol. 23, 301–310. 10.1080/10837450.2017.1329835 28503983

[B206] Tong J. Chen L. (2013). Preparation and application of magnetic chitosan derivatives in separation processes. Anal. Lett. 46, 2635–2656. 10.1080/00032719.2013.807815

[B207] Torchilin V. P. (2008). Tat peptide-mediated intracellular delivery of pharmaceutical nanocarriers. Advanced Drug Delivery Rev. 60, 548–558. 10.1016/j.addr.2007.10.008 18053612

[B208] Tritch R. J. Cheng Y. E. Yin F. H. Erickson-Viitanen S. (1991). Mutagenesis of protease cleavage sites in the human immunodeficiency virus type 1 gag polyprotein. J. Virol. 65, 922–930. 10.1128/JVI.65.2.922-930.1991 1987379 PMC239833

[B209] Tsai W.-B. Chen Y.-R. Liu H.-L. Lai J.-Y. (2011). Fabrication of UV-crosslinked chitosan scaffolds with conjugation of RGD peptides for bone tissue engineering. Carbohydr. Polymers 85, 129–137. 10.1016/j.carbpol.2011.02.003 24750733

[B210] Uto T. Wang X. Sato K. Haraguchi M. Akagi T. Akashi M.. (2007). Targeting of antigen to dendritic cells with poly (γ-glutamic acid) nanoparticles induces antigen-specific humoral and cellular immunity. J. Immunol. 178, 2979–2986. 10.4049/jimmunol.178.5.2979 17312143

[B211] Van Der Lubben I. M. Konings F. A. J. Borchard G. Verhoef J. C. Junginger H. E. (2001a). In vivo uptake of chitosan microparticles by murine Peyer’s patches: visualization studies using confocal laser scanning microscopy and immunohistochemistry. J. Drug Targeting 9, 39–47. 10.3109/10611860108995631 11378522

[B212] Van der Lubben I. M. Verhoef J. C. Borchard G. Junginger H. E. (2001b). Chitosan for mucosal vaccination. Advanced Drug Delivery Rev. 52, 139–144. 10.1016/S0169-409X(01)00197-1 11718937

[B213] Van der Lubben I. M. Verhoef J. C. Van Aelst A. C. Borchard G. Junginger H. E. (2001c). Chitosan microparticles for oral vaccination:: preparation, characterization and preliminary *in vivo* uptake studies in murine Peyer’s patches. Biomaterials 22, 687–694. 10.1016/S0142-9612(00)00231-3 11246962

[B214] van Ginkel F. W. Jackson R. J. Yuki Y. McGhee J. R. (2000). Cutting edge: the mucosal adjuvant cholera toxin redirects vaccine proteins into olfactory tissues. J. Immunol. 165, 4778–4782. 10.4049/jimmunol.165.9.4778 11045998

[B215] Van Reeth K. Ma W. (2012). “Swine influenza virus vaccines: to change or not to change—that’s the question,” in Swine Influenza (Springer). 10.1007/82_2012_266 22976350

[B216] van Riet E. Ainai A. Suzuki T. Hasegawa H. (2012). Mucosal IgA responses in influenza virus infections; thoughts for vaccine design. Vaccine 30, 5893–5900. 10.1016/j.vaccine.2012.04.109 22835738

[B217] Varma A. J. Deshpande S. V. Kennedy J. F. (2004). Metal complexation by chitosan and its derivatives: a review. Carbohydr. Polymers 55, 77–93. 10.1016/j.carbpol.2003.08.005

[B218] Vicente S. Peleteiro M. Díaz-Freitas B. Sanchez A. González-Fernández Á. Alonso M. J. (2013). Co-delivery of viral proteins and a TLR7 agonist from polysaccharide nanocapsules: a needle-free vaccination strategy. J. Control Release 172, 773–781. 10.1016/j.jconrel.2013.09.012 24076340

[B219] Vincent A. L. Ma W. Lager K. M. Janke B. H. Richt J. A. (2008). Swine influenza viruses: a North American perspective. Adv. Virus Res. 72, 127–154. 10.1016/S0065-3527(08)00403-X 19081490

[B220] Vincent A. L. Perez D. R. Rajao D. Anderson T. K. Abente E. J. Walia R. R.. (2017). Influenza A virus vaccines for swine. Vet. Microbiol. 206, 35–44. 10.1016/j.vetmic.2016.11.026 27923501 PMC8609643

[B221] Wang D. Christopher M. E. Nagata L. P. Zabielski M. A. Li H. Wong J. P.. (2004). Intranasal immunization with liposome-encapsulated plasmid DNA encoding influenza virus hemagglutinin elicits mucosal, cellular and humoral immune responses. J. Clin. Virol. 31, 99–106. 10.1016/j.jcv.2004.09.013 15567101

[B222] Wang W. Guo Z. Chen Y. Liu T. Jiang L. (2006). Influence of generation 2-5 of PAMAM dendrimer on the inhibition of Tat peptide/TAR RNA binding in HIV-1 transcription. Chem. Biol. Drug Des. 68, 314–318. 10.1111/j.1747-0285.2006.00454.x 17177893

[B223] Wang Y. Wang X. Luo G. Dai Y. (2008). Adsorption of bovin serum albumin (BSA) onto the magnetic chitosan nanoparticles prepared by a microemulsion system. Bioresource Technol. 99, 3881–3884. 10.1016/j.biortech.2007.08.017 17892932

[B224] Wang J. Feng S. S. Wang S. Chen Z. Y. (2010). Evaluation of cationic nanoparticles of biodegradable copolymers as siRNA delivery system for hepatitis B treatment. Int. J. Pharm. 400, 194–200. 10.1016/j.ijpharm.2010.08.026 20801205

[B225] Wang W. Wang S.-X. Guan H.-S. (2012). The antiviral activities and mechanisms of marine polysaccharides: an overview. Marine Drugs 10, 2795–2816. 10.3390/md10122795 23235364 PMC3528127

[B226] Wang H. Han Q. Zhao H. Xu D. Zhang J. (2018). Single dose HBsAg CS-γ-PGA nanogels induce potent protective immune responses against HBV infection. Eur. J. Pharm. Biopharm. 124, 82–88. 10.1016/j.ejpb.2017.12.003 29247691

[B227] Wang X. Xing B. (2007). Importance of structural makeup of biopolymers for organic contaminant sorption. Environ. Sci. Technol. 41, 3559–3565. 10.1021/es062589t 17547178

[B228] Wedmore I. McManus J. G. Pusateri A. E. Holcomb J. B. (2006). A special report on the chitosan-based hemostatic dressing: experience in current combat operations. J. Trauma Acute Care Surg. 60, 655–658. 10.1097/01.ta.0000199392.91772.44 16531872

[B229] Wong H. L. Chattopadhyay N. Wu X. Y. Bendayan R. (2010). Nanotechnology applications for improved delivery of antiretroviral drugs to the brain. Advanced Drug Delivery Rev. 62, 503–517. 10.1016/j.addr.2009.11.020 19914319

[B230] Wu D. Ensinas A. Verrier B. Primard C. Cuvillier A. Champier G.. (2016). Zinc-Stabilized Chitosan-Chondroitin Sulfate Nanocomplexes for HIV-1 Infection Inhibition Application. Mol. Pharm. 13, 3279–3291. 10.1021/acs.molpharmaceut.6b00568 27454202

[B231] Xiao B. Wan Y. Zhao M. Liu Y. Zhang S. (2011). Preparation and characterization of antimicrobial chitosan-N-arginine with different degrees of substitution. Carbohydr. Polymers 83, 144–150. 10.1016/j.carbpol.2010.07.032

[B232] Yang K. W. Li X. R. Yang Z. L. Li P. Z. Wang F. Liu Y. (2009). Novel polyion complex micelles for liver-targeted delivery of diammonium glycyrrhizinate: In vitro and *in vivo* characterization. J. Biomed. Mater. Res. Part A: Off. J. Soc. Biomater. Japanese Soc. Biomater. Aust. Soc. Biomater. Korean Soc. Biomater. 88, 140–148. 10.1002/jbm.a.31866 18260143

[B233] Yang L. Chen L. Zeng R. Li C. Qiao R. Hu L.. (2010). Synthesis, nanosizing and *in vitro* drug release of a novel anti-HIV polymeric prodrug: chitosan-O-isopropyl-5’-O-d4T monophosphate conjugate. Bioorg. Med. Chem. 18, 117–123. 10.1016/j.bmc.2009.11.013 19959368

[B234] Yang Y. Kuang Y. Yuan D. Cao L. L. Wang B. N. Li M. Y.. (2011). [Investigation of HCV multi-epitope gene vaccine loaded by CTS-Fe3O4]. Sichuan Da Xue Xue Bao Yi Xue Ban 42, 757–61, 88. 22332536

[B235] Yang J. Luo K. Li D. Yu S. Cai J. Chen L.. (2013). Preparation, characterization and *in vitro* anticoagulant activity of highly sulfated chitosan. Int. J. Biol. Macromol. 52, 25–31. 10.1016/j.ijbiomac.2012.09.027 23041665

[B236] Yang Y. Liu Y. Chen S. Cheong K.-L. Teng B. (2020a). Carboxymethyl β-cyclodextrin grafted carboxymethyl chitosan hydrogel-based microparticles for oral insulin delivery. Carbohydr. Polymers 246, 116617. 10.1016/j.carbpol.2020.116617 32747257

[B237] Yang Y. Xing R. Liu S. Qin Y. Li K. Yu H.. (2020b). Chitosan, hydroxypropyltrimethyl ammonium chloride chitosan and sulfated chitosan nanoparticles as adjuvants for inactivated Newcastle disease vaccine. Carbohydr. Polym. 229, 115423. 10.1016/j.carbpol.2019.115423 31826462

[B238] Yen M.-T. Yang J.-H. Mau J.-L. (2009). Physicochemical characterization of chitin and chitosan from crab shells. Carbohydr. Polymers 75, 15–21. 10.1016/j.carbpol.2008.06.006

[B239] Yen H.-L. McKimm-Breschkin J. L. Choy K.-T. Wong D. D. Y. Cheung P. P. H. Zhou J.. (2013). Resistance to neuraminidase inhibitors conferred by an R292K mutation in a human influenza virus H7N9 isolate can be masked by a mixed R/K viral population. MBio 4. 10.1128/mBio.00396-13 PMC3735122 23860768

[B240] You J. Hu F.-Q. Du Y.-Z. Yuan H. (2007). Polymeric micelles with glycolipid-like structure and multiple hydrophobic domains for mediating molecular target delivery of paclitaxel. Biomacromolecules 8, 2450–2456. 10.1021/bm070365c 17661518

[B241] You J. Hu F.-Q. Du Y.-Z. Yuan H. (2008). Improved cytotoxicity of doxorubicin by enhancing its nuclear delivery mediated *via* nanosized micelles. Nanotechnology 19, 255103. 10.1088/0957-4484/19/25/255103 21828645

[B242] Yuki Y. Kiyono H. (2003). New generation of mucosal adjuvants for the induction of protective immunity. Rev. Med. Virol. 13, 293–310. 10.1002/rmv.398 12931340

[B243] Zeng P. Xu Y. Zeng C. Ren H. Peng M. (2011). Chitosan-modified poly(D,L-lactide-co-glycolide) nanospheres for plasmid DNA delivery and HBV gene-silencing. Int. J. Pharm. 415, 259–266. 10.1016/j.ijpharm.2011.05.053 21645597

[B244] Zhai Y. Zhou Y. Li X. Feng G. (2015). Immune-enhancing effect of nano-DNA vaccine encoding a gene of the prME protein of Japanese encephalitis virus and BALB/c mouse granulocyte-macrophage colony-stimulating factor. Mol. Med. Rep. 12, 199–209. 10.3892/mmr.2015.3419 25738258 PMC4438877

[B245] Zhang W. Yin Z. Liu N. Yang T. Wang J. Bu Z.. (2010). DNA-chitosan nanoparticles improve DNA vaccine-elicited immunity against Newcastle disease virus through shuttling chicken interleukin-2 gene. J. Microencapsul 27, 693–702. 10.3109/02652048.2010.507881 21034363

[B246] Zhang N. Wardwell P. R. Bader R. A. (2013). Polysaccharide-based micelles for drug delivery. Pharmaceutics 5, 329–352. 10.3390/pharmaceutics5020329 24300453 PMC3834947

[B247] Zhang E. Xing R. Liu S. Qin Y. Li K. Li P. (2019). Advances in chitosan-based nanoparticles for oncotherapy. Carbohydr. Polymers 222, 115004. 10.1016/j.carbpol.2019.115004 31320066

[B248] Zhao L.-M. Shi L.-E. Zhang Z.-L. Chen J.-M. Shi D.-D. Yang J.. (2011). Preparation and application of chitosan nanoparticles and nanofibers. Braz. J. Chem. Eng. 28, 353–362. 10.1590/S0104-66322011000300001

[B249] Zhao K. Zhang Y. Zhang X. Li W. Shi C. Guo C.. (2014a). Preparation and efficacy of Newcastle disease virus DNA vaccine encapsulated in chitosan nanoparticles. Int. J. Nanomed. 9, 389–402. 10.2147/IJN.S54226 PMC3890423 24426783

[B250] Zhao K. Zhang Y. Zhang X. Shi C. Wang X. Wang X.. (2014b). Chitosan-coated poly(lactic-co-glycolic) acid nanoparticles as an efficient delivery system for Newcastle disease virus DNA vaccine. Int. J. Nanomed. 9, 4609–4619. 10.2147/IJN.S70633 PMC4207079 25356070

[B251] Zhao K. Sun Y. Chen G. Rong G. Kang H. Jin Z.. (2016). Biological evaluation of N-2-hydroxypropyl trimethyl ammonium chloride chitosan as a carrier for the delivery of live Newcastle disease vaccine. Carbohydr. Polym. 149, 28–39. 10.1016/j.carbpol.2016.04.085 27261727

[B252] Zhao K. Li S. Li W. Yu L. Duan X. Han J.. (2017). Quaternized chitosan nanoparticles loaded with the combined attenuated live vaccine against Newcastle disease and infectious bronchitis elicit immune response in chicken after intranasal administration. Drug Delivery 24, 1574–1586. 10.1080/10717544.2017.1388450 29029568 PMC8241129

[B253] Zheng M. Qu D. Wang H. Sun Z. Liu X. Chen J.. (2016). Intranasal administration of chitosan against influenza A (H7N9) virus infection in a mouse model. Sci. Rep. 6, 28729. 10.1038/srep28729 27353250 PMC4926116

[B254] Zoulim F. Perrillo R. (2008). Hepatitis B: reflections on the current approach to antiviral therapy. J. Hepatol. 48, S2–S19. 10.1016/j.jhep.2008.01.011 18304680

